# Lipid A Remodeling Is a Pathoadaptive Mechanism That Impacts Lipopolysaccharide Recognition and Intracellular Survival of Burkholderia pseudomallei

**DOI:** 10.1128/IAI.00360-18

**Published:** 2018-09-21

**Authors:** Michael H. Norris, Nawarat Somprasong, Herbert P. Schweizer, Apichai Tuanyok

**Affiliations:** a Department of Infectious Diseases and Immunology, College of Veterinary Medicine, University of Florida, Gainesville, Florida, USA; b Department of Molecular Genetics and Microbiology, College of Medicine, Emerging Pathogens Institute, University of Florida, Gainesville, Florida, USA; Georgia Institute of Technology School of Biological Sciences

**Keywords:** Burkholderia pseudomallei, inflammasome, intracellular pathogen, lipid A, lipopolysaccharide

## Abstract

Burkholderia pseudomallei causes the severe disease melioidosis. The bacterium subverts the host immune system and replicates inside cells, and host mortality results primarily from sepsis-related complications. Lipopolysaccharide (LPS) is a major virulence factor and mediator of sepsis that many pathogens capable of intracellular growth modify to reduce their immunological “footprint.” The binding strength of B. pseudomallei LPS for human LPS binding protein (hLBP) was measured using surface plasmon resonance. The structures of lipid A isolated from B. pseudomallei under different temperatures were analyzed by matrix-assisted laser desorption ionization–time of flight mass spectrometry (MALDI-TOF MS), and the gene expression of two lipid A remodeling genes, *lpxO* and *pagL*, was investigated. The LPS was characterized for its ability to trigger tumor necrosis factor alpha (TNF-α) release and to activate caspase-11-triggered pyroptosis by introduction of LPS into the cytosol. Lipid A from long-term chronic-infection isolates was isolated and characterized by MALDI-TOF MS and also by the ability to trigger caspase-11-mediated cell death. Lipid A from B. pseudomallei 1026b *lpxO* and *pagL* mutants were characterized by positive- and negative-mode MALDI-TOF MS to ultimately identify their role in lipid A structural modifications. Replication of *lpxO* and *pagL* mutants and their complements within macrophages showed that lipid A remodeling can effect growth in host cells and activation of caspase-11-mediated cytotoxicity.

## Abstract (precis)

Burkholderia pseudomallei causes the severe disease melioidosis. The bacterium subverts the host immune system and replicates inside cells, and host mortality results primarily from sepsis-related complications.

## INTRODUCTION

Mammalian immune systems recognize pathogen-associated molecular patterns (PAMPs) to defend against invading bacteria. Once such PAMP is lipopolysaccharide (LPS). LPS comprises the outer leaflet of the outer membrane of Gram-negative bacteria and is regarded as a major virulence factor. Mammals have a complex set of countermeasures that recognize such an abundant, conserved molecule. The hydrophobic lipid-containing portion of LPS, known as lipid A, is recognized by multiple extracellular components of the innate immune system, including LPS binding protein (LBP), CD14, and Toll-like receptor 4 (TLR4) (1–5). The resulting intracellular signaling cascade serves to quickly activate inflammatory processes that can lead to bacterial lysis, phagocytosis, and the initial stages of adaptive immunity activation (6). More recently, mechanisms involving caspase-11-mediated recognition of intracellular LPS that trigger the inflammasome, leading to pyroptotic cell death, have been characterized (7–9). In these works, it was shown that penta- and hexa-acylated LPS can trigger the inflammasome but tetra-acylated cannot. If LPS overwhelms either pathway, the host response can lead to endotoxic shock and death. Bacterial pathogens have evolved intricate mechanisms that allow successful breaching of host defenses by preventing recognition of their lipid A. Critically, intracellular pathogens must minimize recognition by both pathways for survival within the intracellular niche to protect the bacteria from lysis by antimicrobial compounds (10–12). Bacteria have evolved pathways for modification of lipid A by the addition of 4-amino-arabinose (Ara4N) residues or ethanolamine to phosphate groups (13, 14), removal of acyl chains or phosphate groups (15–20), or lengthening and shortening of acyl chains present in lipid A (12). Yersinia pestis is known to produce the immunologically active hexa-acylated lipid A when present in the intermediate flea host and the temperature is 21°C. When shifted to 37°C, human body temperature, the tetra-acylated form that is weakly immunogenic predominates (21). If engineered to maintain hexa-acylated lipid A at 37°C, the bacteria are rendered avirulent in the mouse model. Francisella tularensis attaches longer acyl chains at 37°C than at lower temperatures, a modification that is essential for virulence (22). Brucella abortus is known to decorate the glucosamine residues with pyrophosphorylethanolamine (23), and Salmonella can add Ara4N residues, add ethanolamine, remove acyl chains, add acyl chains, and hydroxylate acyl chains (13, 17, 24–27). A common theme among successful Gram-negative intracellular pathogens is that they modify lipid A, and that modification can contribute to full virulence.

Burkholderia pseudomallei is a Gram-negative facultative intracellular organism that is found primarily in tropical soil environments, where humans are considered accidental hosts (12). The pathogen can infect most tissues and is considered a biothreat agent. High diversity within the species results in a wide range of virulence. Fifty percent lethal dose (LD_50_) values in the intranasal mouse model can range from 1 × 10^1^ to 1 × 10^6^ CFU (28, 29). LPS is also a site of diversity in the species; serotypes A, A_v_, B, B_v_, B2, and rough have been identified (30–32; unpublished observations). The structure of lipid A in B. pseudomallei and the closely related Burkholderia mallei consists of a parental molecule of penta-acylated lipid A that can contain a single hydroxyl modification of one of the acyl chains and Ara4N modifications (33). Recently, subtle variability in lipid A structures has been described (30). Commingled in the same environment as B. pseudomallei are similar species of bacteria with highly homologous genomes. Burkholderia thailandensis used to be considered an avirulent arabinose-assimilating B. pseudomallei but has since been realized as a separate species with distinct properties (34, 35). A pertinent example is that additional hydroxylation of B. pseudomallei lipid A is not observed in gas chromatography-mass spectrometry (GC-MS) of B. thailandensis lipid A (36). By comparing proinflammatory cytokine release from host cells treated with B. thailandensis LPS to those treated with B. pseudomallei LPS, it was hypothesized that the hydroxylation of B. pseudomallei lipid A results in decreased TLR4 recognition (33). In Salmonella enterica and Bordetella pertussis, the gene responsible for hydroxylation of lipid A was determined to be an aspartyl beta-hydroxylase gene, *lpxO* (15, 27, 37). In S. enterica serovar Typhimurium, derepressed *lpxO* activity is detrimental to efficient invasion of human epithelial cells and to full virulence in the animal model, while in comparison, an *lpxO* null mutation resulted in increased invasion of epithelial cells (38). The primary means by which hydroxylation prevents immune system recognition has not been shown, but hydroxylation is increased when Salmonella is grown at low Mg^2+^ levels and low pH, consistent with the nutrient environment inside phagocytic vesicles (26).

**FIG 1:**
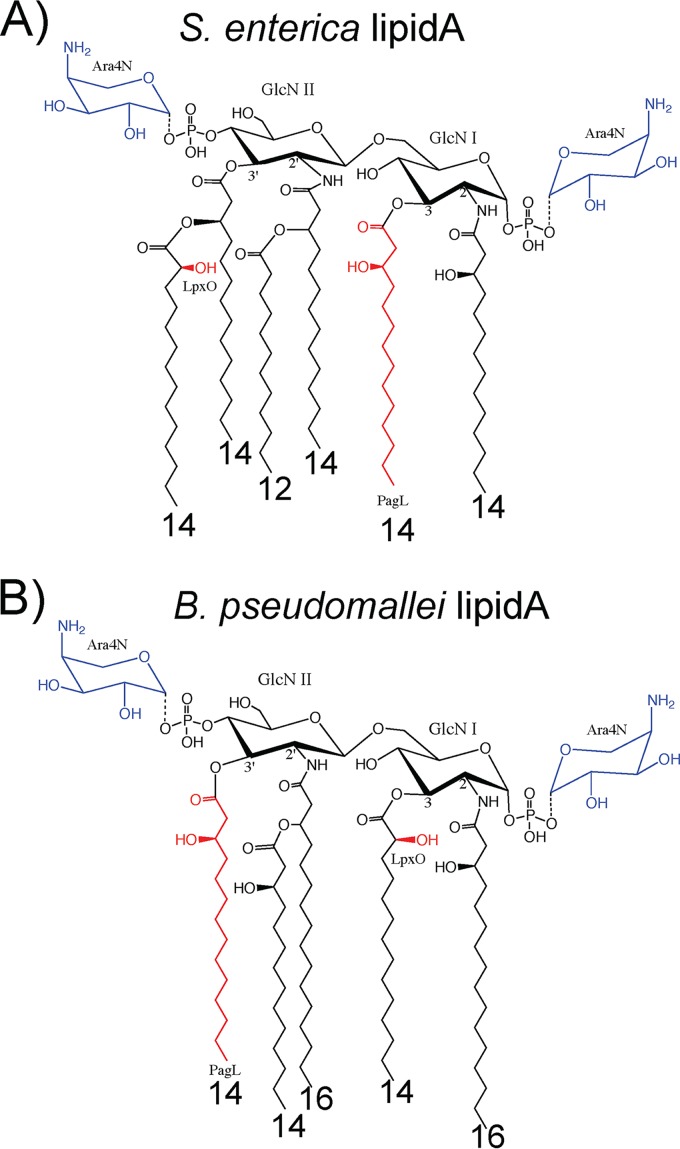
Lipid A structures. (A) S. enterica lipid A. Well-characterized LpxO and PagL modifications are shown in red. Ara4N additions are shown in blue. The structure was reproduced from Kawasaki et al. (40) and Gibbons et al. (52). (B) Current B. pseudomallei lipid A structure from the literature, with assumed PagL and LpxO modifications (30, 33, 43, 51). Carbon lengths of acyl chains are indicated below each chain.

Another gene involved in modifying the parental form of Salmonella lipid A is *pagL*. PagL, characterized as the product of a PhoP activated gene, catalyzes 3-*O*-deacylation of lipid A in the membrane of S. enterica, which is controlled by the PhoP/PhoQ two-component system (39). In S. enterica, PagL is not able to deacylate Ara4N-modified lipid A, so it occurs only when Ara4N modifications are not present (40). Innate immunity recognizes hexa-acylated lipid A most efficiently, and *pagL*-modified lipid A activation of NF-κB through TLR4 is 30- to 100-fold lower than that of unmodified lipid A (17). Decreased acylation may serve a physiological purpose in regulating membrane fluidity, permeability, or outer membrane vesiculation (24, 41) but also renders the abundant LPS less immunogenic. The deacylation of Y. pestis and F. tularensis lipid A at human body temperature exemplifies a pathoadaptive capacity. Tetra-acylated lipid A is frequently observed in lipid A isolated from B. mallei, B. pseudomallei, and B. thailandensis (30, 42, 43) alongside penta-acylated lipid A, so it is likely that a *pagL* homologue exists in B. pseudomallei to enzymatically modify the parental penta-acylated lipid A (Fig. 1).

In our previous work, we found that some strains of B. pseudomallei show different levels of hydroxylation (30). Hydroxylation resulted in decreased innate immunity activation through TLR4. Ultimately, this may lead to decreased inflammation during acute-phase infections. At 24 h postinfection, inflammation in the mouse lung following intranasal challenge with B. pseudomallei was lower when the infecting strain had hydroxylated lipid A and correlated with decreased virulence (44). Of course, pathogens react to increased host defenses by producing virulence factors that damage host cells. Intracellular pathogens utilize similar mechanisms to establish and maintain infections, and in terms of lipid A modification, it is unlikely that B. pseudomallei is the exception to the rule. B. pseudomallei lives primarily in the soil, and humans are not vital to the life cycle. Therefore, the major role for lipid A modification may be physiological, fortuitously providing a survival advantage during stressful growth conditions such as in soil environments, including predation by amoebae or other soilborne eukaryotic organisms.

In this work, we hypothesized that B. pseudomallei modifies its lipid A, resulting in decreased recognition by host immune system pattern recognition receptors (PRRs). Taken together, this work is a significant contribution to understanding the mechanisms and effects of B. pseudomallei lipid A remodeling and the contribution to bacterial immune evasion and intracellular survival.

## RESULTS

### LpxO and PagL in B. pseudomallei.

The *B. pseudomallei lpxO* gene (BP1026B_II1514) encodes a 299-amino-acid (aa), ∼35-kDa protein annotated as a hypothetical protein with probable aspartyl/asparaginyl beta-hydroxylase activity that is 57% identical and 71% homologous at the amino acid level to Salmonella enterica serovar Typhimurium LpxO. It was noticed that 2-hydroxymyristic acid was found only in fatty acid profiles of B. pseudomallei and not in the profiles of avirulent B. thailandensis (36). This led to the identification of the *lpxO* gene in B. pseudomallei K96243 as BPSS1422 and the use of it as a quantitative PCR (qPCR) target for differentiating B. pseudomallei from B. thailandensis (45, 46). It is conspicuously absent in B. thailandensis. The *pagL* gene present in B. pseudomallei has low homology to the S. enterica pagL gene but encodes a hypothetical protein annotated as a lipid A 3-*O*-deacylase containing a PagL domain. B. pseudomallei PagL (BPSL0505 or BP1026B_I2991) is a 189-aa, ∼20-kDa peptide that has 26% identity and 42% homology to Salmonella PagL and is predicted by blastp to be an outer membrane channel family protein. B. pseudomallei PagL contains all conserved catalytic residues mechanistically determined by amino acid substitution (16) and protein crystallography of P. aeruginosa PagL (47). This work also demonstrated the specificity of the binding pocket of PagL for lipid X, which is essentially GlcN I of lipid A. The gene immediately upstream and divergent to *pagL* in B. pseudomallei is *rpoH*, which encodes a heat-responsive sigma factor shown to upregulate heat shock proteins GroEL and HtpG (48). The close proximity to a heat-responsive sigma factor raises the interesting possibility that B. pseudomallei may have a pathoadaptive response to temperature.

### SPR study of B. pseudomallei lipid A binding to hLBP.

**FIG 2:**
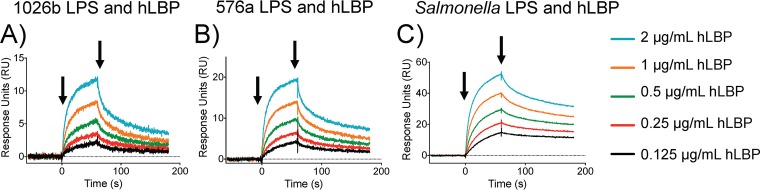
Differences in B. pseudomallei lipid A structure affect binding strength to human LBP. Surface plasmon resonance was used to measure the binding rate constants between 1026b LPS and hLBP (A), 576a LPS and hLBP (B), and Salmonella Minnesota LPS and hLBP (C). Data shown are the raw data average of triplicate experiments. Colored lines represent different concentrations of hLBP, as indicated. Arrows indicate hLBP injection start and injection stop points.

**FIG 3:**
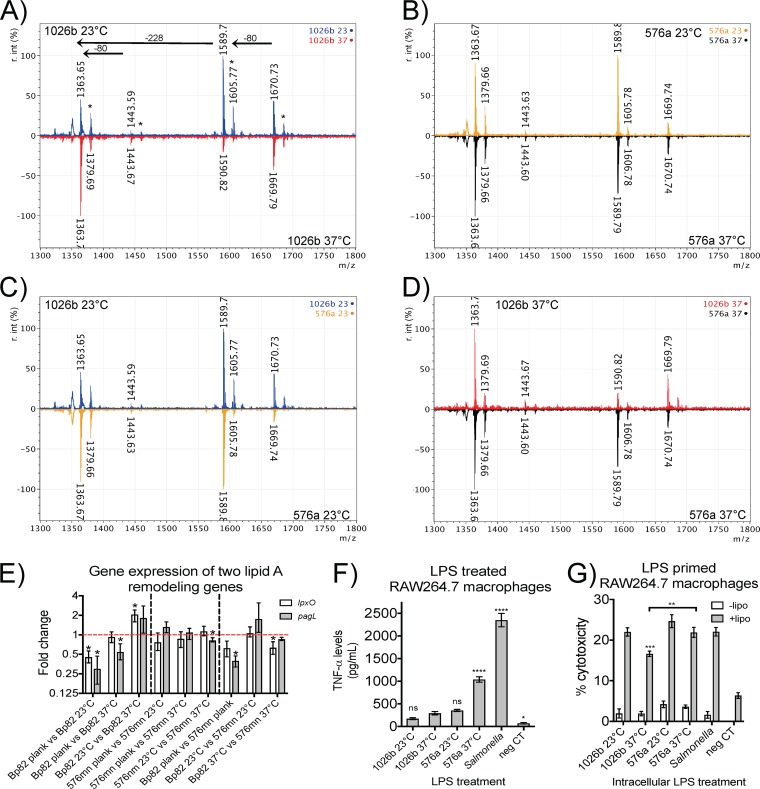
Temperature dependency of lipid A profiles and expression of relevant genes. (A to D) MALDI-TOF MS scans of lipid A show that 1026b lipid A acylation is decreased in response to temperature while 576a lipid A acylation remains the same. It was also found that 576a lipid A hydroxylation was decreased in comparison to that of 1026b. 1026b 23°C lipid A, blue scan; 1026b 37°C lipid A, red scan; 576a 23°C, orange scan; 576a 37°C, black scan. *, –OH peaks; −228, mass of one C_14:0_ acyl chain; −80, mass of one phosphate group. r. int (%), percent relative intensity. (E to G) Gene expression analysis agrees with the observations shown in panels A to D. Scans are the normalized average of at least two LPS preparations. (E) In the 1026b derivative Bp82, *lpxO* and *pagL* had the highest expression in planktonic cells at 37°C, followed by plated bacteria at 37°C, and then plated bacteria at 23°C (with the lowest). The 576a derivative 576mn showed no significant differential gene expression of these two genes. *lpxO* expression in 576mn was 50% that of Bp82 when both were planktonic cells grown at 37°C, while *pagL* expression was significantly lower (∼60% lower). At 23°C, there was little difference between the two, but at 37°C, *lpxO* expression in 576mn was nearly half that in Bp82. Data shown are the standard deviation of ratios resulting from three independent experiments carried out in technical duplicate. Significance was determined using REST software by propagation of efficiencies. (F) TNF-α release from RAW264.7 monolayers following treatment with 10 ng/ml of endotoxin-normalized LPS for 24 h. Significance was determined by one-way ANOVA to 1026b 37°C LPS treatment. neg CT, negative control. (G) Cytotoxicity caused by intracellular triggering of the caspase-11 pyroptotic pathway by 1026b 37°C LPS was significantly lower than that of Salmonella LPS and 576a 37°C LPS. White bars indicate LPS primed and incubated with the indicated LPS but in the absence of Lipofectamine (−lipo), while gray bars represent treatments equivalent to those indicated by the white bars but in the presence of Lipofectamine (+lipo). Significance in inflammasome activation experiments was determined by a one-way ANOVA comparison to Salmonella LPS treatment. *, *P* < 0.05; **, *P* < 0.01; ***, *P* < 0.001; ****, *P* < 0.0001. Data shown are the mean and standard deviation of a representative result of three independent experiments carried out in triplicate.

LBP shuttles LPS in serum to the TLR4-MD2 complex by binding lipid A in a manner similar to that of TLR4-MD2 but with higher affinity. This allows quick LPS recognition when LPS serum concentrations are low but delayed responses when serum concentrations are high, such as during acute-phase infections. Accordingly, surface plasmon resonance (SRP) was used to accurately determine the binding kinetics of the isolated LPS for hLBP. Analyte concentrations and flow conditions were empirically determined in the course of experimental optimization. Empty flow cells were used as negative controls for background subtraction from the flow cells with bound LPS. Salmonella serovar Minnesota S-form LPS served as the positive control. Multiple experiments done in repetition showed that hLBP had a *k_a_* (association constant) ratio of 1.62:1.18:1 (Salmonella:576a:1026b) (Fig. 2A to C and Table 1). Association constants were very similar for the two B. pseudomallei LPSs but about 60% lower than that of Salmonella LPS. The *K_d_* (dissociation constant) of hLBP for the LPS gave a ratio of 0.35:0.73:1 (Salmonella:576a:1026b). The KD values (*K_d_*/*k_a_*) for hLBP bound to the LPS at a ratio of 0.2:0.6:1 (Salmonella:576a:1026b). The equilibrium constant KD values indicate that the hexa-acylated hLBP binds Salmonella LPS with 5 times higher affinity than 1026b LPS, while hLBP binds 576a LPS with almost 2 times higher affinity than 1026b LPS. Once the LPS is immobilized, the differences in O-antigen size do not contribute to measurements in SPR, so the difference in affinity of hLBP for LPS can be directly attributed to the lipid A structural differences seen in Fig. 3D, namely, the tetra-/penta-acylation ratio, the mono-/biphosphorylation ratio, and the degree of hydroxylation.

**TABLE 1:** Binding constants of different LPSs for hLBP as measured by SPR

Source of LPS	*k_a_* (1/Ms)	*k_d_* (1/s)	KD (*k_d_*/*k_a_*) (M)
1026b	1.37E+06	9.64E−03	7.01E−09
576a	1.62E+06	7.00E−03	4.32E−09
Salmonella	2.22E+06	3.41E−03	1.53E−09

### Lipid A masses of B. pseudomallei grown at different temperatures are correlated with differential gene expression of *lpxO* and *pagL*, TNF-α release, and inflammasome activation.

Lipid A of LPS prepared from cells grown on solid medium showed high-quality mass scans and peaks that were consistent with our previous observations. Data acquisition was repeatable and consistent among replicates. Lipid A from B. pseudomallei 1026b grown at 23°C showed biphosphorylated penta-acylated lipid A at 1,670.73 *m/z*, and well-defined hydroxyl peaks 16 *m/z* units higher than the parental molecule are indicated by asterisks in Fig. 3A (blue). The lipid A mass peaks from B. pseudomallei 1026b grown at 37°C (Fig. 3A, red) show an increase in the amount of singly phosphorylated tetra-acyl lipid A and a marked decrease in all penta-acylated species compared to lipid A from B. pseudomallei 1026b grown at 23°C. Lipid A from the serotype B strain B. pseudomallei 576a showed all the characteristic peaks but less hydroxyl modification of penta-acylated lipid A than 1026b and little differences when grown at the two temperatures (Fig. 3B; 576a at 23°C, orange scan; 576a at 37°C, black scan). Figures 3C and D show the conservation of mass peaks and differences in the peak intensity profiles between the two strains when they are grown at the different temperatures. Table S1 in the supplemental material summarizes the lipid A percentages in each sample: the relative percentages of tetra-acylated, penta-acylated, and hydroxylated lipid A.

The modification of lipid A by strain 1026b, but not by 576a, in response to temperature was verified in a gene expression experiment using the select-agent exempt strains of each (44, 49). Genes *lpxO* and *pagL*, which are thought to cause the major lipid A modifications, were targeted for qPCR transcript analysis. RNA was isolated from strains Bp82 and 576mn grown on plates at 23°C and 37°C and also from planktonic cells at 37°C as a control. In Bp82 grown at 23°C, *lpxO* and *pagL* both showed significant downregulation compared to the planktonically grown cells (Fig. 3E). Bacteria grown on plates at 37°C showed no difference in *lpxO* expression from that of the planktonic control but still had significantly decreased expression of *pagL*. Plate-grown Bp82 showed increased expression of both *lpxO* and *pagL* at 37°C compared to 23°C, reflecting the mass peak differences observed in Fig. 3A to D. In comparison, 576mn showed no significant differences in expression under the three conditions tested, again in agreement with the observed mass peaks. Figure 3E also shows that in planktonically grown cells and statically grown cells at 37°C, *lpxO* and *pagL* are expressed at higher levels in Bp82 than in 576mn. Gene expression in the biosafe strains derived from the virulent strains verified our matrix-assisted laser desorption ionization–time of flight mass spectrometry (MALDI-TOF MS) observations.

TNF-α release by RAW264.7 macrophages in response to the various LPSs was measured after 24 h treatment with 10 ng/ml of each LPS that had been normalized to endotoxin units equivalent to Salmonella LPS (Fig. 3F). Endotoxicity was determined by the limulus amebocyte lysate (LAL) assay. The samples in order of increasing TNF-α release were as follows: 1026b at 23°C < 1026b at 37°C < 576a at 23°C ≪ 576a at 37°C ≪ Salmonella. However, results for 1026b at 23°C, 1026b at 37°C, and 576a at 23°C LPS were not significantly different from one another. The observed differences appear to be the result of higher levels of hydroxylation in 1026b LPS. The 576a at 37°C LPS has the largest amounts of doubly phosphorylated penta-acylated LPS of all the samples, slightly higher than the 576a at 23°C sample, combined with the lowest hydroxylation. This indicates that hydroxylation can interfere with formation of the TLR4-MD2-LPS complex.

The different LPSs were tested for their abilities to activate pyroptotic cell death via the caspase-11 intracellular LPS recognition pathway (Fig. 3G). The different LPSs were transfected into the cytoplasm of RAW264.7 macrophages that had been primed overnight with *S*. Minnesota LPS. Transfection of 1 μg of endotoxin unit-normalized LPS samples resulted in cytotoxicity as measured by lactate dehydrogenase (LDH) release. LPS from 1026b grown at 37°C showed significantly lower cytotoxicity than the Salmonella LPS control, while LPS from 1026b grown at 23°C did not. In the absence of transfection reagent, negligible amounts of cytotoxicity were observed, indicating that cell death was due solely to intracellular LPS. Cytotoxicity trends were also consistent with observed mass trends and can be ascribed to the hydroxylation and acylation patterns presented. LPS from 1026b grown at 37°C had the smallest amount of penta-acylated LPS, and that correlated with the lowest observed cytotoxicity in LPS-primed cells.

### Chronic-infection B. pseudomallei lipid A and inflammasome activation.

**FIG 4:**
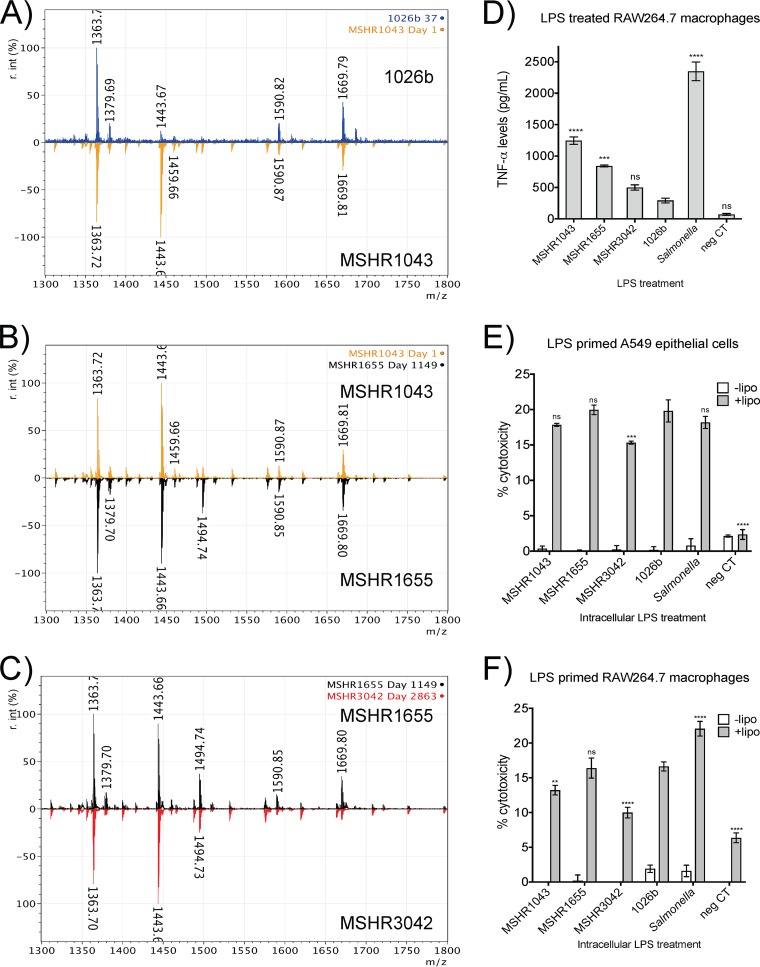
Lipid A structural change in a chronic-infection B. pseudomallei strain series decreases LPS recognition. (A) In comparison to lipid A from 1026b (blue scan), lipid A from MSHR1043 (orange scan) has fewer penta-acylated (5 + 2P and 5 + 1P) structures and hydroxylation (–OH) appears minimal. (B) MSHR1655 (black scan) shows more arabinose-substituted (Ara) tetra-acylated lipid A (1,494 *m/z*) than strain MSHR1043 collected 3 years earlier. (C) MSHR3042 (red scan) had nearly no detectable levels of penta-acylated lipid A compared to strain MSHR1655 collected 5 years earlier. (D) TNF-α release from RAW264.7 monolayers 24 h after treatment with 10 ng/ml of the indicated endotoxin-normalized LPS. (E) Cytotoxicity measurements following LPS transfection into Salmonella LPS-primed A549 human lung epithelial cells. (F) Cytotoxicity measurements following LPS transfection into Salmonella LPS-primed RAW264.7 murine macrophages. White bars represent no-Lipofectamine controls, and gray bars represent exactly the same treatment but with Lipofectamine. Data are the mean and standard deviation of the results of one representative experiment carried out in triplicate. Significance was determined by one-way ANOVA to 1026b. *, *P* < 0.05; **, *P* < 0.01; ***, *P* < 0.001; ****, *P* < 0.0001; ns, not significant.

Strains MSHR1043, MSHR1655, and MSHR3042 were isolated on days 1, 1149, and 2863 from the same patient, patient P314, as previously described (50). The O-antigen is intact in strain MSHR1043, while strains MSHR1655 and MSHR3042 have acquired single-nucleotide polymorphisms (SNPs) that result in the production of rough LPS, as described previously (31). Large-batch LPS isolation from bacteria grown on plates at 37°C and subsequent high-quality MALDI-TOF MS scans show structural alteration of the lipid A over time (Fig. 4A to C). MSHR1043 has high levels of mono- and biphosphorylated tetra-acylated lipid A and low levels of penta-acylated lipid A compared to 1026b (Fig. 4A and Table S2). MSHR1655 lipid A was nearly identical to MSHR1043 lipid A, except for a very prominent peak showing Ara4N modification at ∼1,494 *m/z* (Fig. 4B and Table S2). This modification is associated with a membrane-modifying stress response that leads to increased antimicrobial resistance, potentially attributed to extended antimicrobial treatments experienced by patient P314 during the time between strain isolation. Strain MSHR3042 lipid A is almost entirely tetra-acylated, as evidenced by a near absence of penta-acylated peaks in comparison to scans for strains MSRH1043, MSHR1655, and 1026b (Fig. 4C and Table S2).

The effect of chronic-infection strain LPS lipid A modification on the activation of TLR4 was determined by measuring TNF-α release after treatment with 10 ng/ml of the various LPSs for 24 h (Fig. 4D). The samples in order of increasing TNF-α release were 1026b < MSHR3042 < MSHR1655 < MSHR1043 ≪ Salmonella, although TNF-α levels following 1026b and MSHR3042 LPS treatment were not significantly different. There were very low if any detectable amounts of hydroxylation in the chronic-infection strain LPSs, whereas high levels were present in 1026b. Lack of hydroxylation can be overcome by decreased acylation. These data confirm what is known in the literature, i.e., that lower levels of acylation (as in MSHR3042) can lead to decreased activation of TLR4-triggered cytokine release. Hydroxylation is also a variable pathoadaptive capability not seen in the chronic-infection strains but profoundly affects recognition of B. pseudomallei LPS, as with 1026b LPS.

It is understood that the modifications of lipid A can lead to decreased TLR4 signaling, but we wanted to understand whether the lipid A modifications observed in the chronic-infection strains leads to decreased activation of the inflammasome. Inflammasome-induced cytotoxicity was measured by transfecting the LPSs of these strains directly into the cytoplasm of LPS-primed A549 human lung epithelial cells or RAW264.7 murine macrophages and comparing the results to those of 1026b, Salmonella, and phosphate-buffered saline (PBS) controls. Without transfection reagent, low levels of cytotoxicity were measured, but 1026b and Salmonella LPS controls showed significantly increased intracellular cytotoxicity compared to PBS controls and only in the presence of transfection reagent (Fig. 4E). Cytotoxicity of MSHR1655 LPS was not significantly different from that of either 1026b or Salmonella. However, the MSHR1043 and MSHR3042 LPSs induced significantly less cytotoxicity than the 1026b LPS control. This indicates that the lower the amount of penta-acylated B. pseudomallei LPS present, or in the case of MSHR3042 only tetra-acylated LPS, the lower the recognition of intracellular LPS. MSHR1655 probably induces more cytotoxicity than MSHR1043 because MSHR1655 produces rough LPS while MSHR1043 has an intact O-antigen and still produces penta-acylated lipid A (31). Trends were similar in RAW264.7 cells, where MSHR3042 LPS has a very low capacity to trigger caspase-11-dependent cytotoxicity (Fig. 4F). Data presented are representative of three independent experiments, each carried out in triplicate, with significance determined by one-way analysis of variance (ANOVA) to results for 1026b LPS.

### MALDI-TOF MS of lipid A from *B. pseudomallei lpxO* and *pagL* insertional mutants and inflammasome activation by their LPSs.

**FIG 5:**
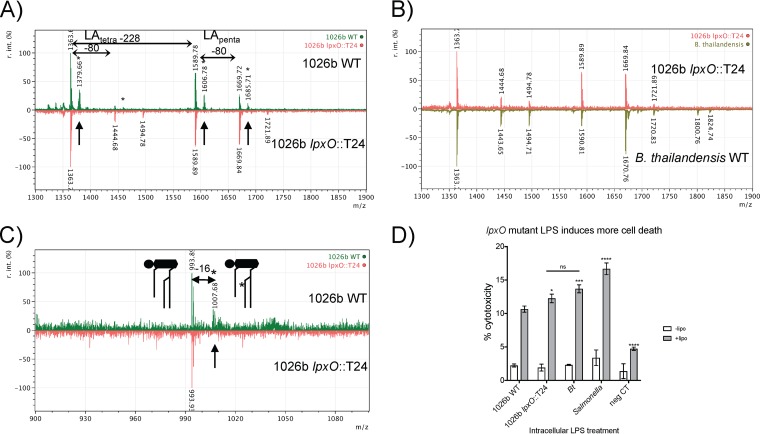
The *B. pseudomallei lpxO* mutant is unable to produce hydroxylated lipid A. (A) MALDI-TOF MS scan in negative-ion mode of 1026b wild-type (WT) lipid A (green scan) compared to that of the *lpxO* mutant (orange scan). Asterisks indicate –OH group peaks, and arrows indicate –OH peaks missing from the *lpxO* mutant lipid A scan. Horizontal arrows indicate 228 mass differences indicative of C_14:0_-OH loss and an 80 mass difference indicative of a phosphate group. (B) The negative-ion mode *B. pseudomallei lpxO* mutant scan (orange scan) compared to the B. thailandensis wild type (green scan). (C) The positive-ion mode scan of the 1026b wild type (green) compared to that of the *lpxO* mutant (orange) from 900 to 1,100 *m/z*, with the only prominent peak of the sodium adduct of the triacylated oxonium ion depicted by the diagram. Mass differences of the –OH are indicated by −16 and the asterisk. The –OH peak indicated by the arrow is absent in the *lpxO* mutant. (D) Cytotoxicity measurements following LPS transfection into Salmonella LPS-primed RAW264.7 murine macrophages. White bars represent no-Lipofectamine controls, and gray bars represent exactly the same treatment but with Lipofectamine. Data are the mean and standard deviation of the results of one representative experiment carried out in triplicate. Significance was determined by one-way ANOVA to 1026b WT LPS or as indicated by a horizontal bar. *, *P* < 0.05; ***, *P* < 0.001; ****, *P* < 0.0001; ns, not significant.

Details of the B. pseudomallei lipid A structure are not refined. Some structural predictions even omit well-characterized mechanisms of lipid A modification due to assumptions of MALDI-TOF MS peaks (30, 42, 43, 51). To put some of these structural assumptions to rest, detailed negative and positive MALDI-TOF MS scans of *lpxO* and *pagL* mutants were acquired. MALDI-TOF MS scans of wild-type 1026b lipid A in the negative mode clearly show the –OH peaks located 16 *m/z* higher than each of the major tetra- and pent-acylated mass peaks (Fig. 5A; in green, indicated by asterisks). These peaks are unmistakably absent in the scan of the lipid A isolated from the *lpxO*::T24 mutant (Fig. 5A, orange scan). In fact, the *lpxO*::T24 mutant scan looks identical to typical B. thailandensis scans (Fig. 5B). The mechanism and result of LpxO modification have been elucidated in Salmonella and demonstrate that the –OH modification occurs on the C_14:0_ esterified to the amide-linked 3-hydroxypalmitate at position 2′ of the GlcN II residue of the disaccharide lipid A backbone (37, 52). Many papers still show the –OH modification of B. pseudomallei lipid A occurring on the ester-linked C_14:0_ at position 3 of the GlcN I residue of lipid A (30, 33, 43, 51). MALDI-TOF MS scans of the 1026b wild type in the positive mode clearly show hydroxylation occurring on the sodium adduct of the phosphorylated triacylated GlcN II oxonium ion of the wild type (green), which is absent in the *lpxO*::T24 mutant scan (orange, indicated by arrow), in agreement with LpxO mechanisms characterized in Salmonella (Fig. 5C). This is in agreement with the certainty that the only open site available on the lipid A for variable hydroxylation by this mechanism is located on the C_14:0_ esterified to the amide-linked 3-hydroxypalmitate at the 3′ position. Inflammasome activation of the *lpxO* mutant LPS in RAW264.7 cells was found to be slightly (but significantly) higher than that of 1026b LPS but was not significantly different from that of B. thailandensis LPS (Fig. 5D and Table S3). This indicates that *lpxO*-mediated hydroxylation of B. pseudomallei lipid A contributes to decreased intracellular LPS recognition of LPS by the caspase-11 inflammasome.

**FIG 6:**
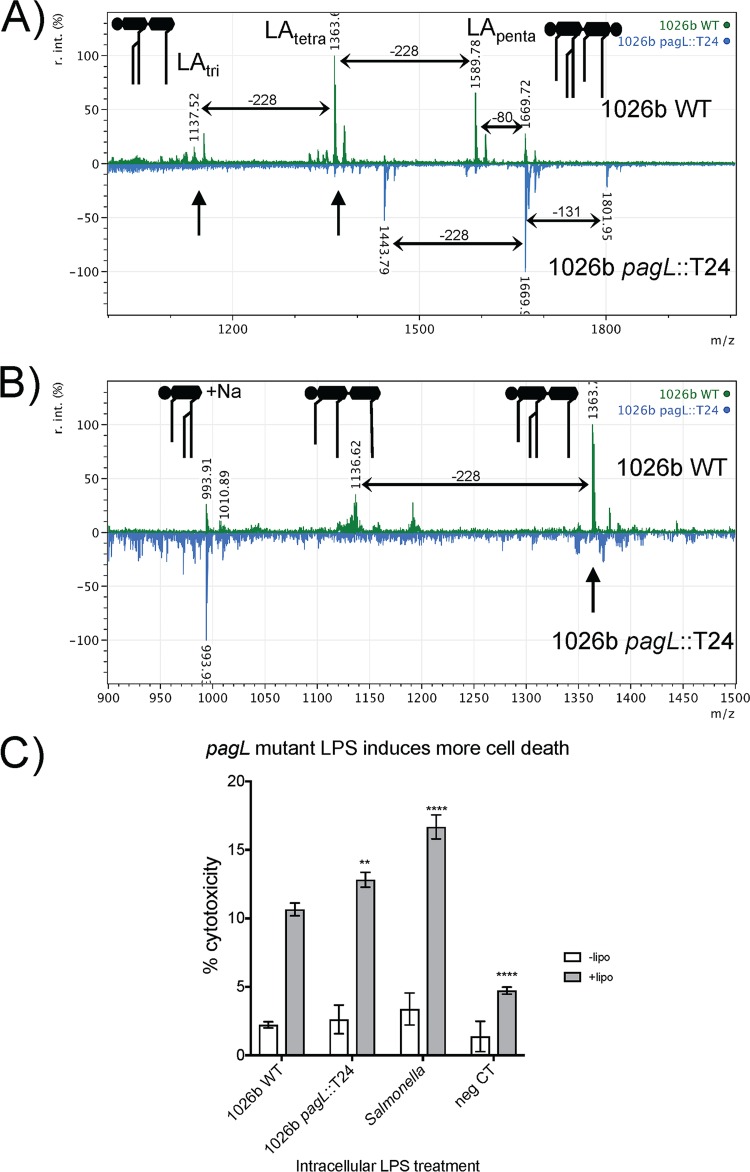
The *B. pseudomallei pagL* mutant is unable to produce tetra-acylated lipid A. (A) Negative-ion mode scan from 1,000 to 2,000 *m/z* of 1026b wild-type lipid A (green) compared to that of the *pagL* mutant (light blue). The diagram shows the simplified penta-acylated (LA_penta_) and triacylated (LA_tri_) lipid A observed. Horizontal arrows indicate −228 mass differences indicative of C_14:0_-OH, a −131 mass difference indicative of amino arabinose, and a −80 mass difference indicative of a phosphate group. (B) Positive-ion mode scan of the same lipid A from 900 to 1,500 *m/z*. (C) Cytotoxicity measurements following LPS transfection into Salmonella LPS-primed RAW264.7 murine macrophages. White bars represent no-Lipofectamine controls, and gray bars represent exactly the same treatment but with Lipofectamine. Data are the mean and standard deviation of the results of one representative experiment carried out in triplicate. Significance was determined by one-way ANOVA to 1026b wild-type LPS. **, *P* < 0.01; ****, *P* < 0.0001.

The function of PagL in the deacylation of lipid A has also been well characterized in Salmonella (16, 17, 24, 25, 47, 53). Ambiguity has been introduced in the literature showing deacylation at the 3′ position of GlcN II by an enzyme with lipid A 3-*O*-deacylase activity in Burkholderia spp. (30, 33, 42, 43, 51). The lipid A 3-*O*-deacylase cannot deacylate from the 3′ position of GlcN II. It has been shown that PagL is a lipid A 3-*O*-deacylase, and crystal structures have been used to identify the mechanism of action (16, 17, 47). MALDI-TOF MS of Burkholderia cenocepacia lipid A revealed tetra- and penta-acylated mass peaks identical to those seen in B. pseudomallei and B. mallei (reference 54 and present study). Detailed nuclear magnetic resonance (NMR) analysis of B. cenocepacia in the same study showed that a majority of the lipid A is tetra-acylated and that position 3 of GlcN I (not position 3′ of GlcN II) is predominantly not acylated. To lay this issue to rest, negative-mode MALDI-TOF MS showed the loss of tetra-acylated peaks in the *pagL* mutant (blue scan indicated by arrow) and an increase in penta-acylated lipid A expected when deacylation occurs (Fig. 6A and Table S3), confirming that *pagL* is needed for deacylation and that penta-acylated lipid A is the parental lipid A molecule. Additional analysis of the fragmentation patterns can be accomplished when armed with the knowledge that only acyl chain substituted at the 3 or 3′ positions can be readily cleaved by MS in the negative mode (55). Furthermore, under constant power, the position 3-linked acyl chains will be cleaved at the lowest energy, followed by the position 3′-linked acyl chains (56). Both 1026b wild type and the *pagL* mutant have penta-acylated lipid A that can be fragmented by removal of the position 3-linked C_14:0_ (3-OH). However, there is no tetra-acylated lipid A in the *pagL* mutant that can be cleaved to triacylated lipid A, so the triacylated peaks due to fragmentation of the position 3′-linked acyl chain from the tetra-acylated lipid A (found at ∼1,137 *m/z*) in the wild type are not observed in the mutant. Positive-mode MALDI-TOF MS was utilized to pinpoint which GlcN residue was deacylated, because fragmentation in the positive mode preferentially occurs at the ester-linked fatty acid substituent of the amide-linked 2′-C_16:0_ (55), resulting in a peak at ∼1,136 *m/z*. As can be seen in Fig. 6B, tetra-acylated peaks are not present in the *pagL* mutant scan indicated by the arrow. Further fragmentation of the tetra-acylated lipid A is possible only in the wild type and is discernibly missing in the mutant due to the absence of PagL activity. Cleavage of the lipid A disaccharide backbone results in the sodium adduct of the phosphorylated triacylated GlcN II oxonium ion at 993.91 *m/z* in both wild-type and *pagL* mutant lipid A. Besides the tri- and tetra-acylated peaks, there were no differences between the wild type and the mutant in peak profiles from 700 to 2,000 *m/z*. Complete similarity at lower *m/z* values indicates that *pagL* is not involved in the deacylation of GlcN II but probably of GlcN I. This is in accordance with the well-characterized Salmonella and Pseudomonas aeruginosa mechanisms of PagL-mediated deacylation, the predicted function of the B. pseudomallei homologue, the conserved active-site residues present in B. pseudomallei PagL, and the previously published B. cenocepacia NMR data. Besides maintaining all conserved active-site residues with P. aeruginosa PagL and B. cenocepacia PagL, B. pseudomallei PagL is 68% identical and 79% similar to the B. cepacia complex homologue. Inflammasome-activated cytotoxicity by *pagL* modification of 1026b LPS was determined in RAW264.7 macrophages (Fig. 6C). LPS from the *pagL* mutant induced significantly higher levels of cytotoxicity than wild-type LPS. The *pagL* mutant produces more penta-acylated lipid A than the wild type (Fig. 6A), which produces a mixture of tetra- and penta-acylation. This makes sense, because tetra-acylated LPS is not strongly recognized by the caspase-11 inflammasome.

**FIG 7:**
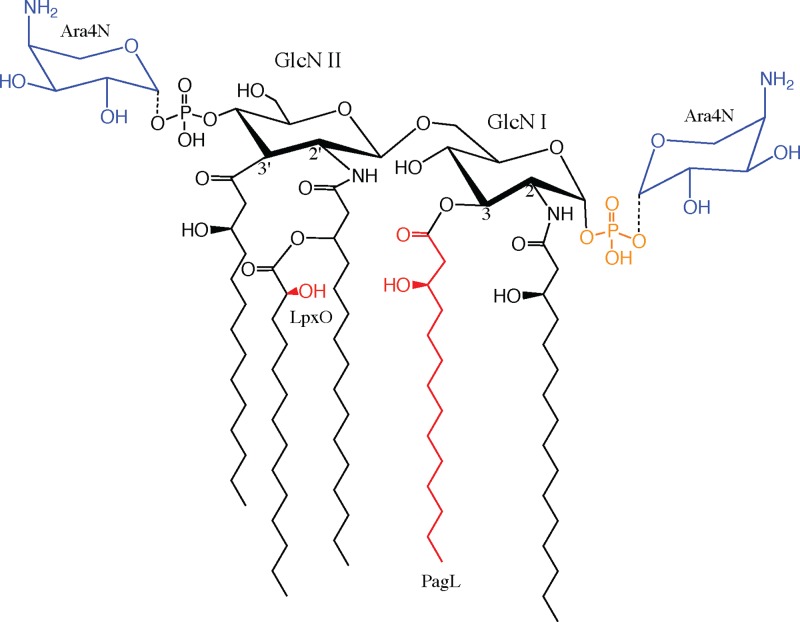
B. pseudomallei lipid A structure. The structures and modifications are based on *lpxO* and *pagL* mutant analysis from a detailed dissection of the literature and this work. Blue residues are Ara4N that are nonstoichiometric, and the orange residue is the phosphate group that is absent in monophosphorylated lipid A. The red hydroxyl group indicates the LpxO modification, and the red acyl chain indicates the PagL modification.

These experiments demonstrate the lipid A modifications by *lpxO* and *pagL* and their role in decreasing the intracellular recognition of LPS. Mass scans of the complemented *lpxO* and *pagL* mutants showed a return of hydroxylation and deacylation to the respective mutant strains (Fig. S1). These findings support the structural determination of the parental B. pseudomallei penta-acylated lipid A and its site-specific modification by LpxO and PagL shown in Fig. 7.

### Effect of *lpxO* and *pagL* mutation on intracellular inflammasome activation, cellular invasion, and intracellular replication.

**FIG 8:**
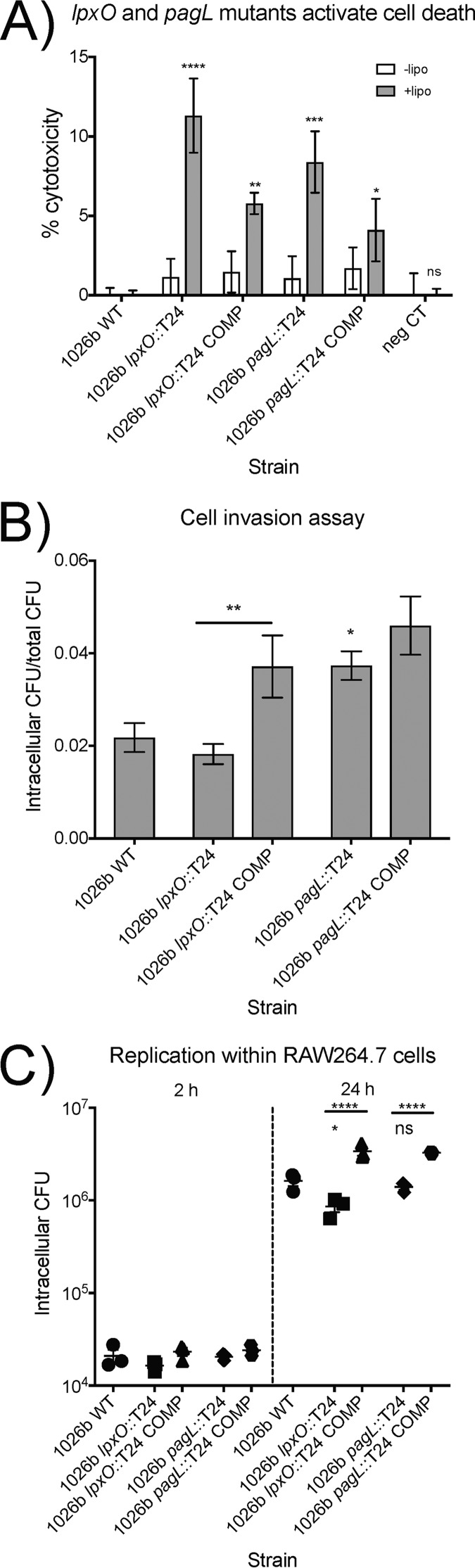
Inflammasome activation, invasion, and intracellular replication by *lpxO* and *pagL* mutants. (A) Cytotoxicity measurements following intracellular infection of unprimed or Salmonella LPS-primed RAW264.7 murine macrophages. White bars represent unprimed macrophage cytotoxicity levels, and gray bars represent cytotoxicity levels of macrophages treated exactly the same but primed overnight with Salmonella LPS. Data are the mean and standard deviation of the results of one representative experiment carried out in triplicate. Significance was determined by one-way ANOVA to the 1026b wild type (WT). (B) Invasiveness of 1026b and the *lpxO* and *pagL* mutants and complements (COMP) in RAW264.7 murine macrophages. (C) Replication of 1026b wild-type bacteria compared to that of the *lpxO* and *pagL* mutants and their complements at 2 and 24 h postinfection. Data shown are the mean and standard deviation of the results of one of two independent experiments carried out in triplicate. Significance was determined by one-way ANOVA comparison to 1026b WT or as indicated by horizontal bars. *, *P* < 0.05; **, *P* < 0.01; ***, *P* < 0.001; ****, *P* < 0.0001; ns, not significant.

The mutants and complements were used to infect unprimed and LPS-primed RAW264.7 macrophages at a multiplicity of infection (MOI) of 10:1. Four hours later, the cytotoxicity was measured and compared to that of the wild type (Fig. 8A). It is immediately apparent that, in this model 4 h after infection, there is no measurable cytotoxicity in 1026b-infected cells regardless of the primed state. At this MOI of 10:1, intracellular 1026b bacteria were not significant activators of the intracellular inflammasome. As intracellular *lpxO* mutant bacteria entered the cytoplasm, they triggered significant cytotoxicity (∼10%). The complemented *lpxO* mutant had reduced cytotoxicity compared to the mutant but not as low as the wild type. Intracellular *pagL* mutant bacteria were also significantly increased in their ability to trigger inflammasome-mediated cytotoxicity (∼7.5%). Similar to the *lpxO* complement, the *pagL* complement showed reduced cytotoxicity compared to the mutant, but cytotoxicity was not reduced to the levels of the wild type.

RAW264.7 macrophages were used to determine if LpxO-mediated hydroxylation or PagL-mediated deacylation of B. pseudomallei affects bacterial invasion. The mutants and complemented strain were used to infect cells at an MOI of 10:1. The amounts of intracellular bacteria at 2 h postinfection are presented as a fraction of the total introduced (Fig. 8B). The *lpxO* mutant had slightly reduced invasion efficiency compared to the wild type, but the complemented *lpxO* mutant strain had nearly double the efficiency, a significant increase over both the wild type and the mutant. It is possible that by complementing via single-copy complementation, we decoupled *lpxO* expression from repressive elements, leading to enhanced hydroxylation of lipid A. Indeed, analysis of the lipid A mass spectrum of the complemented *lpxO* mutant by MALDI-TOF MS showed that the complement had ∼40% more hydroxylated lipid A than the wild type (Fig. S1 and Table S3). This means that *lpxO* contributes to the invasion of macrophages in this model. However, the *pagL* mutant showed higher levels of invasion than the wild type, and the complement showed even higher levels. More bacteria inside the macrophage can stem from two possibilities: (i) enhanced uptake of bacteria or (ii) enhanced bacterial survival. First, elimination of PagL-mediated deacylation, as in the *pagL* mutant, increases the number of intracellular bacteria, because the larger amount of penta-acylated lipid A strongly activates the macrophages' phagocytic ability. Second, the higher activity of PagL and the increased deacylation in the complemented *pagL* mutant allow B. pseudomallei to survive the internalization without activating macrophage defenses. The complement had ∼17% more tetra-acylated lipid A than the wild type and ∼87% more than the *pagL* mutant (Fig. S1 and Table S3). The data imply that *pagL* is important for macrophage invasion and requires precise regulation to maintain the intracellular lifestyle. The invasion trends are reciprocal to the cytotoxicity trends, confirming that the caspase-11-activated inflammasome can partially control intracellular infection but that *lpxO* and *pagL* modifications contribute to the enhanced invasion survival in B. pseudomallei.

Intracellular replication of the strain panel was measured at 2 and 24 h in RAW264.7 macrophages (Fig. 8C). Intracellular numbers were not significantly different at 2 h, even though invasion efficiencies showed differences. At 24 h, the mean number of intracellular CFU of the *lpxO* mutant was ∼50% of that of the wild type, a significant reduction, while that of the *pagL* mutant was ∼86% that of the wild type. As with the invasion assay, complementation resulted in significantly higher numbers of intracellular CFU than those of their respective mutants. The increased invasiveness of the *pagL* mutant did not translate to higher levels of intracellular bacteria at 24 h. The most logical conclusion, based on the results shown in Fig. 8A, is that enhanced macrophage activation led to the control of intracellular bacterial CFU. These data clearly show that the *lpxO*-mediated hydroxylation of B. pseudomallei lipid A is important for replication in the established RAW264.7 macrophage model and that *pagL* plays a less significant role but a role nonetheless.

## DISCUSSION

In this work, we strived to understand the processes that shape lipid A structure in B. pseudomallei. Strain-specific responses to stimuli can cause transcriptional and posttranscriptional modifications that affect lipid A biosynthesis and remodeling. It is a dynamic process that, given enough selective pressure, could become permanent through genomic mutation. We have provided compelling evidence that B. pseudomallei possesses the genes for lipid A modification and that they can be differentially regulated in response to temperature and under different growth conditions (planktonic versus static) and can result in modified lipid A structures that have various effects on host recognition of the molecule.

Furthermore, B. pseudomallei LPS recognition processes upstream and downstream of TLR4 have not received much attention. This work demonstrated that different lipid A profiles can affect interactions with hLBP by directly measuring the affinity of B. pseudomallei LPS for hLBP using SPR. LBP is an important part of the acute-phase systemic innate response to LPS. During infection, an impaired ability to detect LPS can stymie the host response and prevent bacterial clearance. It has been shown that tetra-acylated F. tularensis LPS cannot bind LBP, failing to activate the human polymorphonuclear leukocytes important for early infection control (57). It was found by analysis of intracellular LPS-initiated cell death that the same lipid A structures that have low binding to hLBP and TLR4 are the least efficient at activating caspase-11, as caspase-11 does not responds well to tetra-acylated LPS. The reduction in the cytotoxicity level of B. pseudomallei 1026b LPS at 23°C from that at 37°C may seem modest compared to that for Y. pestis, where hexa-acylated lipid A from bacteria grown at 25°C can trigger ∼50% cytotoxicity and tetra-acylated lipid A from bacteria grown at 37°C is reduced to ∼5% cytotoxicity (8). However, what is reported in this work is similar but opposite to the observed differences in cytotoxicity due to temperature-dependent lipid A modifications in Francisella: ∼20% at 37°C and ∼12% at 18°C (8, 12). It must be kept in mind that B. pseudomallei does not produce hexa-acylated LPS and the shift to tetra-acylated LPS is not complete, as is observed in Y. pestis. B. pseudomallei is probably more similar in this regard to Francisella, where multiple stepwise modifications produce poorly immunogenic lipid A regardless of the condition and which can be made even less immunogenic under the right circumstances. It is unclear if other lipid A modifications affect recognition by caspase-11. This compounding effect means that some B. pseudomallei LPS is poorly recognized at all stages of host defense and may allow for successful host infection. Other B. pseudomallei strains initially establish infections similarly but cause higher levels of inflammation later due to increased proinflammatory cytokine production and increased activation of inflammasome-mediated cytotoxicity during systemic infection. It was also found that long-term chronic carriage of B. pseudomallei in a patient can result in shifts to less immunogenic tetra-acylated LPS. It is well known that decreased acylation leads to less extracellular TLR4 signaling. By extending our analysis to intracellular recognition of LPS, we found that a lack of penta-acylated LPS from long-term chronic-infection isolate MSHR3042 resulted in 20 to 30% less inflammasome-initiated cell death than that caused by 1026b wild-type LPS, depending on the cell line.

Importantly, we were able to identify the genes that B. pseudomallei utilizes to hydroxylate and deacylate its lipid A. Inactivation of *lpxO* abolished the production of hydroxylated lipid A and reproduced a B. thailandensis-like lipid A in B. pseudomallei. Inactivation of *pagL* caused a large shift to penta-acylated lipid A and decreased levels of dephosphorylation. It may be that *pagL* interacts with or is required for efficient lipid A 1-phosphatase activity. There was no B. pseudomallei homologue to known lipid A 1-phosphatases identified by BLAST, but our data imply that a protein of similar function surely exists. Our previously published data indicate that a strain with less hydroxylation induces more TNF-α during acute lung infection and the purified LPS induces a stronger inflammatory response in macrophages and human peripheral blood mononuclear cell (PBMCs) (30, 44). Hydroxylation may be another mechanism to control the level of host recognition of lipid A. LpxO and PagL also have effects on B. pseudomallei invasion and replication in macrophages, presumably by decreasing the activation of phagocyte defenses at the onset of phagocytosis and during intracellular replication.

The same PAMPs that are recognized by extracellular PRRs such as TLRs are recognized by intracellular mechanisms. B. pseudomallei is capable of intracellular replication within host cells, but the saprophytic nature of the organism implies that mechanisms of mammalian pathogenesis evolved in the soil environment. LPS enhances the degradation of phagocytized bacteria in amoebae such as Dictyostelium discoideum (58), and primitive mechanisms exist for bacterial recognition and uptake (59). It stands to reason that the ability to decrease LPS recognition would increase survival during predation and would be a strong selective pressure for B. pseudomallei in soil.

The complex interaction between the two lipid A modifications (*lpxO* and *pagL*) and the two host lipid A detection systems (extracellular and intracellular) contributes to rapid progression to sepsis in the host. Since the host is unable to efficiently detect and eliminate B. pseudomallei, the bacteria grow profusely. Relentless LPS priming and pyroptosis-induced cell death then cause overwhelming sepsis. The effect of *lpxO* and *pagL* mutations on B. pseudomallei pathogenesis is unknown, and our future research will endeavor to determine their contribution. The pathoadaptive nature of B. pseudomallei lipid A modification is another means by which B. pseudomallei evades immune detection and secures its intracellular niche during infection.

## MATERIALS AND METHODS

### Bacterial strains, cell lines, and growth conditions.

All select agent work was carried out in a CDC/USDA tier 1 approved facility at the University of Florida in accordance with tier 1 regulations. All protocols were approved by the Institutional Biosafety Committee prior to implementation. B. pseudomallei strains (CDC/USDA registered in-house bacterial inventory) were cultivated in Lennox broth (5 g/liter NaCl) (LB; Fisher BioReagents) or tryptic soy agar (TSA; Becton Dickinson) and grown at 37°C or 23°C where indicated. LB was used for liquid growth of all strains. LB supplemented with 1,000 μg/ml kanamycin (Km; Fisher Scientific) was used for the selection of mutants and complements in B. pseudomallei strains. B. pseudomallei strain details are as follows. The common LPS type A strain 1026b is considered a low-virulence strain, and the LPS type B strain 576a is considered a high-virulence strain. Strain 1026b was isolated from a nonfatal case of septicemic melioidosis and has an LD_50_ by intraperitoneal (i.p.) injection in BALB/c mice of 5.1 × 10^4^ CFU (60). Strain 576a was isolated from a fatal disseminated melioidosis case and has an LD_50_ of 80 CFU in the i.p. mouse model (61). Both strains are from Thailand, and a significant body of work has been generated for each. Select agent-excluded strains Bp82 (49) and 576mn (44) were grown on LB or TSA with 0.6 mM adenine (Amresco). Human cell line A549 and murine cell line RAW264.7 (American Type Culture Collection [ATCC]) were grown in Dulbecco's modified Eagle medium (DMEM)-high glucose plus l-glutamine (HyClone) with 10% fetal bovine serum (FBS; HyClone) in 5% CO_2_ at 37°C. All plasticware was Corningware with a CellBIND surface. Culturing cells was carried out essentially as described previously (62–65).

### LPS isolation and lipid A preparation.

A modified hot-phenol extraction was utilized to extract LPS from select agent-excluded and select agent B. pseudomallei strains. This was done essentially as described previously (30, 66) but with modifications, validation, and verification included for biosafety level 3 (BSL-3) activities. Each bacterial strain was grown on 8 to 10 plates of TSA for 48 h at 37°C or 72 h at 23°C. Bacterial lawns were delicately flooded with phosphate-buffered saline (PBS) and scraped off using a plate spreader. The bacterial suspensions were aliquoted into 2-ml gasketed microcentrifuge tubes and heat killed at 110°C for 15 min. Phenol was added to the lysed solution to a final concentration of 50%, and 10% of the resulting mixture was washed three times in PBS and plated on LB to ensure sterility. Upon verification of sterility, the samples were moved to BSL-2. All phases were combined and dialyzed using tubing with a 12- to 14-kDa molecular weight cutoff against distilled water for 3 to 5 days until free of phenol. Samples were checked for the presence of LPS by silver staining. Dialyzed samples were treated with DNase I for 2 h, RNase H for 2 h, and proteinase K overnight and then further purified as previously described (66). After lyophilization, dry samples were resuspended in 6 ml of 20 mM sodium acetate (NaOAc) and then spun for 8 h at 4°C and 100,000 × *g* in an ultracentrifuge. The samples were resuspended in 2 ml of endotoxin-free water and lyophilized to determine the dry weight. Samples were compared to S-form LPS from Salmonella Minnesota (Hycult) that was quality controlled for undetectable TLR4-independent activity using the LAL assay (Thermo Scientific Pierce).

To prepare lipid A, 10 mg of LPS was dried under vacuum and resuspended in 0.5 ml of 1 M acetic acid and heated to 100°C for 1.5 h to hydrolyze the lipid A from the core and O-antigen. The sample was dried under vacuum and resuspended in 100 μl Milli-Q water and sonicated for 10 min in a bath-sonicator. Five hundred microliters of acidified ethanol was added to the sample and briefly vortexed. The lipid A was then pelleted by centrifugation at 10,000 × *g* at 4°C for 10 min. The pellet was sonicated, resuspended in 100% ethanol, and centrifuged to wash. Washing with nonacidified ethanol was repeated a total of three times. The pellet was dried under vacuum.

### SPR experiments.

Surface plasmon resonance (SPR) assays were carried out as follows. LPS samples were biotinylated using the EZ-Link NHS-biotin kit according to the manufacturer's recommendations (Thermo Fisher). Biotinylated LPS samples were immobilized on the sensor chip SA using a Biacore T100 to ∼320 response units (RU) after 60 s with HBS-EP+ (GE Healthcare) buffer as the flow buffer. After significant optimization, the ideal flow buffer composition was determined to be HBS-EP+ buffer plus 0.125 mg/ml BSA with an ideal flow rate of 30 μl/min. For each experiment, recombinant hLBP (R&D Systems) was resuspended in flow buffer, and through multiple titration curves, the optimal starting concentration was 2 μg/ml hLBP (39.22 nM) and was diluted serially down to 0.125 μg/ml (2.451 nM). Raw data were analyzed in the Biacore evaluation module, and the curves were fitted to the kinetic model using the Marquart-Levenburg algorithm. Residuals were within error limits for the model. Runs were carried out in triplicate in the kinetic affinity mode. Data are representative of two independent experiments.

### RNA isolation and qPCR.

SYBR green-based qPCR assays were used to measure *lpxO* and *pagL* expression in bacteria grown as described above for LPS isolation and compared to planktonic growth in tryptic soy broth (TSB) with shaking at 225 rpm and 37°C for 12 h. Bacterial lawns and cultures were adjusted to an optical density at 600 nm (OD_600_) of 1 in PBS and then pelleted by centrifugation. Supernatant was removed, and the pellets were resuspended in RNAprotect cell reagent solution. The cells were stored at −80°C until further use. RNA was isolated using the RNeasy minikit (Qiagen) according to the manufacturer's protocol, and mRNA integrity was determined by agarose gel visualization and UV absorbance measurements. RNA was isolated from three experiments, and cDNA was produced using the Superscript III first-strand synthesis system (Thermo Fisher) according to the manufacturer's protocols. Primer sets were designed on the Integrated DNA Technologies real-time PCR tool. Primers used were *lpxO*-F (5′-GCCATTCTGTTCATCTTCGTC-3′), *lpxO*-R (5′-CATAGGCGAAACAATTGAGCG-3′), *pagL*-F (5′-GCAATTCGGCAATCGTCAG-3′), and *pagL*-R (5′-CAGGATTCGGGTCTTTGATACC-3′). Efficiency determination and melt-curve analysis of primer pairs were carried out prior to qPCR by using serially diluted genomic DNA preparations as the template. B. pseudomallei 1026b 16S rRNA genes were chosen as the housekeeping gene to accurately normalize RNA amounts in bacteria grown under different conditions. The cDNA was diluted 1:10 and then cycle thresholds (*C_T_*) were captured on a Bio-Rad CFX-96 using SsoAdvanced universal SYBR green supermix (Bio-Rad). qPCR was done in technical duplicates and biological triplicates with a total of six *C_T_* values for each sample. *C_T_* and efficiency data were exported and analyzed using the REST software suite that takes into account efficiencies of qPCR targets and housekeeping genes using the Pfaffl method of relative gene expression calculation (67). The associated errors can be propagated in this software, giving more accurate significance determinations.

### MALDI-TOF MS analysis of lipid A.

For MALDI-TOF MS analysis, dried samples were solubilized in 100 μl of a chloroform-methanol solution (2:1, vol/vol), and then dilutions of 1:10, 1:100, and 1:1,000 were prepared. To prepare the samples, 10 μl of each dilution was mixed with 10 μl of matrix (500 mM 2,5-dihydrobenzoic acid [42, 68]) and 3 μl of ultrapure water and mixed, and 0.5 μl of each preparation was spotted on a MALDI-TOF plate. The MALDI-TOF MS plate was dried at room temperature. Mass spectrometry data were acquired in a model 4700 MALDI-TOF/TOF analyzer (AB Sciex, Framingham, MA, USA) with 4000 Explorer v3.0 software and a Nd:YAG (neodymium-doped yttrium aluminum garnet) laser with a 200-Hz sampling rate. The instrument was operated in the positive or negative ion reflector mode, using a fixed laser intensity of 3,500 to accumulate 1,000 shots/spectrum, across the mass ranges indicated. Acquired spectra were then converted to mzXML files and analyzed on the open-source mMass software. Lipid A substituent percentages were calculated by dividing the relative peak intensity by the sum of relative peak intensities in the sample as measured by negative-mode MALDI-TOF MS and then multiplying by 100.

### TNF-α release assays.

RAW264.7 macrophages were seeded in 96-well CellBIND plates and treated with 10 ng/ml of endotoxin-normalized LPS samples. After 24 h, the supernatant was diluted 1:10 in DMEM and TNF-α levels were determined using the mouse TNF-α Quantikine ELISA kit as recommended by the manufacturer (R&D Systems, Minneapolis, MN, USA). Optical densities at 450 nm minus the 540-nm backgrounds were measured, and TNF-α levels (in pg/ml) were determined by comparison to a standard curve generated using the four-parameter logistic curve-fit in the Prism Software.

### Inflammasome activation assays.

Inflammasome activation assays were carried out by seeding the RAW264.7 murine macrophage or A549 human epithelial cell lines in 96-well CellBIND plates and allowing them to attach overnight. At the same time, cells were primed with Salmonella Minnesota LPS (Hycult) at 5 μg/ml in DMEM–10% FBS overnight (16 h). Previous works utilized LPS from Salmonella rough mutants (Re-LPS), which is essentially pure lipid A with the addition of two Kdo (3-deoxy-d-manno-octulosonic acid) (8). We wanted to demonstrate that the full molecules are capable of achieving biologically meaningful results. LPS samples were prepared for transfection by mixing 0.25 μl of Lipofectamine in 2 μl of DMEM–0% FBS, which was then mixed with 2 μl of 500 μg/ml (1 μg total) LPS–DMEM–0% FBS for a total of 4 μl. The mixture was allowed to incubate at room temperature for 30 min. Volumes were brought up to 50 μl with DMEM–0% FBS and then used to replace the Salmonella LPS priming medium after three washes with DMEM–0% FBS. Cells were incubated for 6 h (RAW264.7) or 24 h (A549), and then the medium was removed and cytotoxicity was measured using the LDH cytotoxicity assay kit (Thermo Scientific Pierce) according to the manufacturer's specifications.

### Flp-*FRT*-mediated excision of chromosomal resistance markers and complementation of the *lpxO* and *pagL* mutants.

For Flp-*FRT*-mediated excision, plasmid pFlpe2 (69) was electroporated into B. pseudomallei strains prepared as previously described (70) by washing with water and plated on LB–2,000 μg/ml Zeocin (Invitrogen)–0.2% rhamnose and incubated for 48 h at 30°C (69). After 48 h, colonies were purified on LB and incubated at 42°C for 24 h to cure the plasmid. Kanamycin (Flp-*FRT* excision) and Zeocin (plasmid curing) sensitivities were verified by patching purified colonies on LB, LB–2,000 μg/ml zeocin, and LB–1,000 μg/ml kanamycin. Single-copy complementation vector cloning was achieved by EcoRI/HindIII cloning of the 1,432-bp PCR product of *lpxO* (using primers *lpxO*_Up_EcoRI, 5′-GAACCGAATTCGGCGTTCGCGGTT-3′, and *lpxO*_Dn_HindIII, 5′-ACATTAAGCTTCCCGACGCCAAT-3′) and the 953-bp PCR product of *pagL* (using *pagL*_Up_EcoRI, 5′-GGGGGAATTCCTCGATGAATCTT-3′, and *pagL*_Dn_HindIII, 5′-AATAAGCTTCGCCACCATCGCCATAC-3′) amplified from B. pseudomallei 1026b genomic DNA (gDNA) into pUC18T-mini-Tn*7*T-Km-*FRT* (69) cut with the same enzymes. Complementation of markerless mutants by coelectroporation of complementation constructs and the helper plasmid, pTNS3, was verified by PCR of the *glmS*-linked *att*Tn*7* insertion sites, as previously described (69). Complemented mutants with insertions at the *glmS*2 site were used for further characterization.

### Inflammasome activation by bacteria, invasion, and replication assays.

Bacteria were grown for 16 h in LB broth with shaking at 37°C. The OD_600_ was measured and adjusted to 1, and then the mixture was diluted to an MOI of 10:1. Exact numbers of CFU were determined by dilution plating the inoculum. Bacteria were used to infect PBS-washed RAW264.7 monolayers that had been seeded overnight in 24-well CellBIND plates for 1 h. After 1 h, the monolayers were washed with PBS three times. DMEM containing amikacin at 150 μg/ml was added. Monolayers were incubated for 1 h for invasion assays, the medium was removed, and monolayers were washed three times with PBS and then lysed in 0.2% Triton X-100 (vol/vol) in PBS for 15 min. Serial dilutions were made and plated on LB agar and incubated at 37°C for 24 h. Colonies were counted 24 h later and divided by the inoculum plate counts to determine the invasion efficiency. Experiments were carried out in triplicate, and data are representative of two independent experiments.

For inflammasome activation assays, cells were either unprimed or primed overnight with 5 μg/ml of Salmonella LPS and infected the same as described for the invasion and replication assays. At 4 h postinfection (3 h after the addition of amikacin), the supernatant was removed and cytotoxicity was measured by LDH release as described above.

## Supplementary Material

Supplemental file 1

Supplemental file 2

Supplemental file 3

Supplemental file 4

Supplemental file 5

## References

[1] Poltorak A, He X, Smirnova I, Liu MY, Van Huffel C, Du X, Birdwell D, Alejos E, Silva M, Galanos C, Freudenberg M, Ricciardi-Castagnoli P, Layton B, Beutler B 1998 Defective LPS signaling in C3H/HeJ and C57BL/10ScCr mice: mutations in Tlr4 gene. Science 282:2085–2088. doi:10.1126/science.282.5396.2085. 9851930

[2] Muta T, Takeshige K 2001 Essential roles of CD14 and lipopolysaccharide-binding protein for activation of toll-like receptor (TLR)2 as well as TLR4 reconstitution of TLR2- and TLR4-activation by distinguishable ligands in LPS preparations. Eur J Biochem 268:4580–4589. doi:10.1046/j.1432-1327.2001.02385.x. 11502220

[3] Park BS, Song DH, Kim HM, Choi B-S, Lee H, Lee J-O 2009 The structural basis of lipopolysaccharide recognition by the TLR4-MD-2 complex. Nature 458:1191–1195. doi:10.1038/nature07830. 19252480

[4] Deng M, Scott MJ, Loughran P, Gibson G, Sodhi C, Watkins S, Hackam D, Billiar TR 2013 Lipopolysaccharide clearance, bacterial clearance, and systemic inflammatory responses are regulated by cell type-specific functions of TLR4 during sepsis. J Immunol 190:5152–5160. doi:10.4049/jimmunol.1300496. 23562812 PMC3644895

[5] Park BS, Lee J-O 2013 Recognition of lipopolysaccharide pattern by TLR4 complexes. Exp Mol Med 45:e66. doi:10.1038/emm.2013.97. 24310172 PMC3880462

[6] Barrio L, Saez de Guinoa J, Carrasco YR 2013 TLR4 signaling shapes B cell dynamics via MyD88-dependent pathways and Rac GTPases. J Immunol 191:3867–3875. doi:10.4049/jimmunol.1301623. 23997213

[7] Aachoui Y, Leaf IA, Hagar JA, Fontana MF, Campos CG, Zak DE, Tan MH, Cotter PA, Vance RE, Aderem A, Miao EA 2013 Caspase-11 protects against bacteria that escape the vacuole. Science 339:975–978. doi:10.1126/science.1230751. 23348507 PMC3697099

[8] Hagar JA, Powell DA, Aachoui Y, Ernst RK, Miao EA 2013 Cytoplasmic LPS activates caspase-11: implications in TLR4-independent endotoxic shock. Science 341:1250–1253. doi:10.1126/science.1240988. 24031018 PMC3931427

[9] Thurston TL, Matthews SA, Jennings E, Alix E, Shao F, Shenoy AR, Birrell MA, Holden DW 2016 Growth inhibition of cytosolic *Salmonella* by caspase-1 and caspase-11 precedes host cell death. Nat Commun 7:13292. doi:10.1038/ncomms13292. 27808091 PMC5097160

[10] Band VI, Weiss DS 2015 Mechanisms of antimicrobial peptide resistance in Gram-negative bacteria. Antibiotics (Basel) 4:18–41. doi:10.3390/antibiotics4010018. 25927010 PMC4410734

[11] Korneev KV, Arbatsky NP, Molinaro A, Palmigiano A, Shaikhutdinova RZ, Shneider MM, Pier GB, Kondakova AN, Sviriaeva EN, Sturiale L, Garozzo D, Kruglov AA, Nedospasov SA, Drutskaya MS, Knirel YA, Kuprash DV 2015 Structural relationship of the lipid A acyl groups to activation of murine Toll-like receptor 4 by lipopolysaccharides from pathogenic strains of *Burkholderia mallei*, *Acinetobacter baumannii*, and *Pseudomonas aeruginosa*. Front Immunol 6:595. doi:10.3389/fimmu.2015.00595. 26635809 PMC4655328

[12] Li Y, Powell DA, Shaffer SA, Rasko DA, Pelletier MR, Leszyk JD, Scott AJ, Masoudi A, Goodlett DR, Wang X, Raetz CR, Ernst RK 2012 LPS remodeling is an evolved survival strategy for bacteria. Proc Natl Acad Sci U S A 109:8716–8721. doi:10.1073/pnas.1202908109. 22586119 PMC3365160

[13] Trent MS, Ribeiro AA, Lin S, Cotter RJ, Raetz CR 2001 An inner membrane enzyme in *Salmonella* and *Escherichia coli* that transfers 4-amino-4-deoxy-l-arabinose to lipid A: induction on polymyxin-resistant mutants and role of a novel lipid-linked donor. J Biol Chem 276:43122–43131. doi:10.1074/jbc.M106961200. 11535604

[14] Zhou Z, Ribeiro AA, Lin S, Cotter RJ, Miller SI, Raetz CR 2001 Lipid A modifications in polymyxin-resistant *Salmonella typhimurium*: PmrA-dependent 4-amino-4-deoxy-l-arabinose, and phosphoethanolamine incorporation. J Biol Chem 276:43111–43121. doi:10.1074/jbc.M106960200. 11535603

[15] MacArthur I, Jones JW, Goodlett DR, Ernst RK, Preston A 2011 Role of *pagL* and *lpxO* in *Bordetella bronchiseptica* lipid A biosynthesis. J Bacteriol 193:4726–4735. doi:10.1128/JB.01502-10. 21764941 PMC3165656

[16] Geurtsen J, Steeghs L, Hove JT, van der Ley P, Tommassen J 2005 Dissemination of lipid A deacylases (PagL) among gram-negative bacteria: identification of active-site histidine and serine residues. J Biol Chem 280:8248–8259. doi:10.1074/jbc.M414235200. 15611102

[17] Kawasaki K, Ernst RK, Miller SI 2004 3-*O*-deacylation of lipid A by PagL, a PhoP/PhoQ-regulated deacylase of *Salmonella typhimurium*, modulates signaling through Toll-like receptor 4. J Biol Chem 279:20044–20048. doi:10.1074/jbc.M401275200. 15014080

[18] Wang X, McGrath SC, Cotter RJ, Raetz CR 2006 Expression cloning and periplasmic orientation of the *Francisella novicida* lipid A 4′-phosphatase LpxF. J Biol Chem 281:9321–9330. doi:10.1074/jbc.M600435200. 16467300 PMC2758525

[19] Wang X, Karbarz MJ, McGrath SC, Cotter RJ, Raetz CR 2004 MsbA transporter-dependent lipid A 1-dephosphorylation on the periplasmic surface of the inner membrane: topography of *Francisella novicida* LpxE expressed in *Escherichia coli*. J Biol Chem 279:49470–49478. doi:10.1074/jbc.M409078200. 15339914 PMC2552400

[20] Tran AX, Whittimore JD, Wyrick PB, McGrath SC, Cotter RJ, Trent MS 2006 The lipid A 1-phosphatase of *Helicobacter pylori* is required for resistance to the antimicrobial peptide polymyxin. J Bacteriol 188:4531–4541. doi:10.1128/JB.00146-06. 16740959 PMC1482963

[21] Kawahara K, Tsukano H, Watanabe H, Lindner B, Matsuura M 2002 Modification of the structure and activity of lipid A in *Yersinia pestis* lipopolysaccharide by growth temperature. Infect Immun 70:4092–4098. doi:10.1128/IAI.70.8.4092-4098.2002. 12117916 PMC128165

[22] Montminy SW, Khan N, McGrath S, Walkowicz MJ, Sharp F, Conlon JE, Fukase K, Kusumoto S, Sweet C, Miyake K, Akira S, Cotter RJ, Goguen JD, Lien E 2006 Virulence factors of *Yersinia pestis* are overcome by a strong lipopolysaccharide response. Nat Immunol 7:1066–1073. doi:10.1038/ni1386. 16980981

[23] Casabuono AC, Czibener C, Del Giudice MG, Valguarnera E, Ugalde JE, Couto AS 2017 New features in the lipid A structure of *Brucella suis* and *Brucella abortus* lipopolysaccharide. J Am Soc Mass Spectrom 28:2716–2723. doi:10.1007/s13361-017-1805-x. 28924631

[24] Kawasaki K, Manabe T 2010 Latency of the lipid A deacylase PagL is involved in producing a robust permeation barrier in the outer membrane of *Salmonella enterica*. J Bacteriol 192:5837–5840. doi:10.1128/JB.00758-10. 20833808 PMC2953691

[25] Manabe T, Kawasaki K 2008 Extracellular loops of lipid A 3-*O*-deacylase PagL are involved in recognition of aminoarabinose-based membrane modifications in *Salmonella enterica* serovar Typhimurium. J Bacteriol 190:5597–5606. doi:10.1128/JB.00587-08. 18567660 PMC2519386

[26] Gibbons HS, Kalb SR, Cotter RJ, Raetz CR 2005 Role of Mg^2+^ and pH in the modification of *Salmonella* lipid A after endocytosis by macrophage tumour cells. Mol Microbiol 55:425–440. doi:10.1111/j.1365-2958.2004.04409.x. 15659161

[27] Guo L, Lim KB, Gunn JS, Bainbridge B, Darveau RP, Hackett M, Miller SI 1997 Regulation of lipid A modifications by *Salmonella typhimurium* virulence genes *phoP-phoQ*. Science 276:250–253. doi:10.1126/science.276.5310.250. 9092473

[28] Barnes JL, Ketheesan N 2005 Route of infection in melioidosis. Emerg Infect Dis 11:638–639. doi:10.3201/eid1104.041051. 15834987 PMC3320332

[29] Ulett GC, Currie BJ, Clair TW, Mayo M, Ketheesan N, Labrooy J, Gal D, Norton R, Smith CA, Barnes J, Warner J, Hirst RG 2001 *Burkholderia pseudomallei* virulence: definition, stability and association with clonality. Microbes Infect 3:621–631. doi:10.1016/S1286-4579(01)01417-4. 11445448

[30] Norris MH, Schweizer HP, Tuanyok A 2017 Structural diversity of *Burkholderia pseudomallei* lipopolysaccharides affects innate immune signaling. PLoS Negl Trop Dis 11:e0005571. doi:10.1371/journal.pntd.0005571. 28453531 PMC5425228

[31] Tuanyok A, Stone JK, Mayo M, Kaestli M, Gruendike J, Georgia S, Warrington S, Mullins T, Allender CJ, Wagner DM, Chantratita N, Peacock SJ, Currie BJ, Keim P 2012 The genetic and molecular basis of O-antigenic diversity in *Burkholderia pseudomallei* lipopolysaccharide. PLoS Negl Trop Dis 6:e1453. doi:10.1371/journal.pntd.0001453. 22235357 PMC3250505

[32] Burtnick MN, Brett PJ, Woods DE 2002 Molecular and physical characterization of *Burkholderia mallei* O antigens. J Bacteriol 184:849–852. doi:10.1128/JB.184.3.849-852.2002. 11790757 PMC139525

[33] Novem V, Shui G, Wang D, Bendt AK, Sim SH, Liu Y, Thong TW, Sivalingam SP, Ooi EE, Wenk MR, Tan G 2009 Structural and biological diversity of lipopolysaccharides from *Burkholderia pseudomallei* and *Burkholderia thailandensis*. Clin Vaccine Immunol 16:1420–1428. doi:10.1128/CVI.00472-08. 19692625 PMC2756838

[34] Brett PJ, DeShazer D, Woods DE 1998 *Burkholderia thailandensis* sp. nov., description of *Burkholderia pseudomallei*-like species. Int J Syst Bacteriol 48:317–320. doi:10.1099/00207713-48-1-317. 9542103

[35] Smith MD, Angus BJ, Wuthiekanun V, White NJ 1997 Arabinose assimilation defines a nonvirulent biotype of *Burkholderia pseudomallei*. Infect Immun 65:4319–4321. 9317042 10.1128/iai.65.10.4319-4321.1997 PMC175618

[36] Inglis TJ, Aravena-Roman M, Ching S, Croft K, Wuthiekanun V, Mee BJ 2003 Cellular fatty acid profile distinguishes *Burkholderia pseudomallei* from avirulent *Burkholderia thailandensis*. J Clin Microbiol 41:4812–4814. doi:10.1128/JCM.41.10.4812-4814.2003. 14532228 PMC254375

[37] Gibbons HS, Lin S, Cotter RJ, Raetz CR 2000 Oxygen requirement for the biosynthesis of the *S*-2-hydroxymyristate moiety in *Salmonella typhimurium* lipid A. Function of LpxO, a new Fe^2+^/alpha-ketoglutarate-dependent dioxygenase homologue. J Biol Chem 275:32940–32949. 10903325 10.1074/jbc.M005779200

[38] Moreira CG, Herrera CM, Needham BD, Parker CT, Libby SJ, Fang FC, Trent MS, Sperandio V 2013 Virulence and stress-related periplasmic protein (VisP) in bacterial/host associations. Proc Natl Acad Sci U S A 110:1470–1475. doi:10.1073/pnas.1215416110. 23302685 PMC3557018

[39] Trent MS, Pabich W, Raetz CR, Miller SI 2001 A PhoP/PhoQ-induced lipase (PagL) that catalyzes 3-*O*-deacylation of lipid A precursors in membranes of *Salmonella typhimurium*. J Biol Chem 276:9083–9092. doi:10.1074/jbc.M010730200. 11108722

[40] Kawasaki K, Ernst RK, Miller SI 2005 Inhibition of *Salmonella enterica* serovar Typhimurium lipopolysaccharide deacylation by aminoarabinose membrane modification. J Bacteriol 187:2448–2457. doi:10.1128/JB.187.7.2448-2457.2005. 15774888 PMC1065228

[41] Elhenawy W, Bording-Jorgensen M, Valguarnera E, Haurat MF, Wine E, Feldman MF 2016 LPS remodeling triggers formation of outer membrane vesicles in *Salmonella*. mBio 7:e00940-16. doi:10.1128/mBio.00940-16. 27406567 PMC4958258

[42] Brett PJ, Burtnick MN, Snyder DS, Shannon JG, Azadi P, Gherardini FC 2007 *Burkholderia mallei* expresses a unique lipopolysaccharide mixture that is a potent activator of human Toll-like receptor 4 complexes. Mol Microbiol 63:379–390. doi:10.1111/j.1365-2958.2006.05519.x. 17163980 PMC1974782

[43] Weehuizen TA, Prior JL, van der Vaart TW, Ngugi SA, Nepogodiev SA, Field RA, Kager LM, van 't Veer C, de Vos AF, Wiersinga WJ 2015 Differential Toll-like receptor-signalling of *Burkholderia pseudomallei* lipopolysaccharide in murine and human models. PLoS One 10:e0145397. doi:10.1371/journal.pone.0145397. 26689559 PMC4687033

[44] Norris MH, Rahman Khan MS, Schweizer HP, Tuanyok A 2017 An avirulent *Burkholderia pseudomallei* Δ*purM* strain with atypical type B LPS: expansion of the toolkit for biosafe studies of melioidosis. BMC Microbiol 17:132. doi:10.1186/s12866-017-1040-4. 28592242 PMC5461690

[45] Merritt A, Inglis TJJ, Chidlow G, Harnett G 2006 PCR-based identification of *Burkholderia pseudomallei*. Rev Inst Med Trop São Paulo 48:239–244. 17086309 10.1590/s0036-46652006000500001

[46] Kaestli M, Richardson LJ, Colman RE, Tuanyok A, Price EP, Bowers JR, Mayo M, Kelley E, Seymour ML, Sarovich DS, Pearson T, Engelthaler DM, Wagner DM, Keim PS, Schupp JM, Currie BJ 2012 Comparison of TaqMan PCR assays for detection of the melioidosis agent *Burkholderia pseudomallei* in clinical specimens. J Clin Microbiol 50:2059–2062. doi:10.1128/JCM.06737-11. 22442327 PMC3372170

[47] Rutten L, Geurtsen J, Lambert W, Smolenaers JJ, Bonvin AM, de Haan A, van der Ley P, Egmond MR, Gros P, Tommassen J 2006 Crystal structure and catalytic mechanism of the LPS 3-*O*-deacylase PagL from *Pseudomonas aeruginosa*. Proc Natl Acad Sci U S A 103:7071–7076. doi:10.1073/pnas.0509392103. 16632613 PMC1564273

[48] Vanaporn M, Vattanaviboon P, Thongboonkerd V, Korbsrisate S 2008 The *rpoE* operon regulates heat stress response in *Burkholderia pseudomallei*. FEMS Microbiol Lett 284:191–196. doi:10.1111/j.1574-6968.2008.01216.x. 18507684

[49] Propst KL, Mima T, Choi KH, Dow SW, Schweizer HP 2010 A *Burkholderia pseudomallei* Δ*purM* mutant is avirulent in immunocompetent and immunodeficient animals: candidate strain for exclusion from select-agent lists. Infect Immun 78:3136–3143. doi:10.1128/IAI.01313-09. 20404077 PMC2897367

[50] Price EP, Sarovich DS, Mayo M, Tuanyok A, Drees KP, Kaestli M, Beckstrom-Sternberg SM, Babic-Sternberg JS, Kidd TJ, Bell SC, Keim P, Pearson T, Currie BJ 2013 Within-host evolution of *Burkholderia pseudomallei* over a twelve-year chronic carriage infection. mBio 4:e00388-13. doi:10.1128/mBio.00388-13. 23860767 PMC3735121

[51] Sengyee S, Yoon SH, Paksanont S, Yimthin T, Wuthiekanun V, Limmathurotsakul D, West TE, Ernst RK, Chantratita N 2018 Comprehensive analysis of clinical *Burkholderia pseudomallei* isolates demonstrates conservation of unique lipid A structure and TLR4-dependent innate immune activation. PLoS Negl Trop Dis 12:e0006287. doi:10.1371/journal.pntd.0006287. 29474381 PMC5842036

[52] Gibbons HS, Reynolds CM, Guan Z, Raetz CR 2008 An inner membrane dioxygenase that generates the 2-hydroxymyristate moiety of *Salmonella* lipid A. Biochemistry 47:2814–2825. doi:10.1021/bi702457c. 18254598 PMC2709818

[53] Kawasaki K, China K, Nishijima M 2007 Release of the lipopolysaccharide deacylase PagL from latency compensates for a lack of lipopolysaccharide aminoarabinose modification-dependent resistance to the antimicrobial peptide polymyxin B in *Salmonella enterica*. J Bacteriol 189:4911–4919. doi:10.1128/JB.00451-07. 17483225 PMC1913436

[54] Silipo A, Molinaro A, Cescutti P, Bedini E, Rizzo R, Parrilli M, Lanzetta R 2005 Complete structural characterization of the lipid A fraction of a clinical strain of *B. cepacia* genomovar I lipopolysaccharide. Glycobiology 15:561–570. doi:10.1093/glycob/cwi029. 15610978

[55] Sforza S, Silipo A, Molinaro A, Marchelli R, Parrilli M, Lanzetta R 2004 Determination of fatty acid positions in native lipid A by positive and negative electrospray ionization mass spectrometry. J Mass Spectrom 39:378–383. doi:10.1002/jms.598. 15103651

[56] Crittenden CM, Akin LD, Morrison LJ, Trent MS, Brodbelt JS 2017 Characterization of Lipid A variants by energy-resolved mass spectrometry: impact of acyl chains. J Am Soc Mass Spectrom 28:1118–1126. doi:10.1007/s13361-016-1542-6. 27966172 PMC5438766

[57] Barker JH, Weiss J, Apicella MA, Nauseef WM 2006 Basis for the failure of *Francisella tularensis* lipopolysaccharide to prime human polymorphonuclear leukocytes. Infect Immun 74:3277–3284. doi:10.1128/IAI.02011-05. 16714555 PMC1479269

[58] Walk A, Callahan J, Srisawangvong P, Leuschner J, Samaroo D, Cassilly D, Snyder ML 2011 Lipopolysaccharide enhances bactericidal activity in *Dictyostelium discoideum* cells. Dev Comp Immunol 35:850–856. doi:10.1016/j.dci.2011.03.018. 21527280 PMC3131744

[59] Chen G, Zhuchenko O, Kuspa A 2007 Immune-like phagocyte activity in the social amoeba. Science 317:678–681. doi:10.1126/science.1143991. 17673666 PMC3291017

[60] Welkos SL, Klimko CP, Kern SJ, Bearss JJ, Bozue JA, Bernhards RC, Trevino SR, Waag DM, Amemiya K, Worsham PL, Cote CK 2015 Characterization of *Burkholderia pseudomallei* strains using a murine intraperitoneal infection model and *in vitro* macrophage assays. PLoS One 10:e0124667. doi:10.1371/journal.pone.0124667. 25909629 PMC4409376

[61] Atkins T, Prior RG, Mack K, Russell P, Nelson M, Oyston PC, Dougan G, Titball RW 2002 A mutant of *Burkholderia pseudomallei*, auxotrophic in the branched chain amino acid biosynthetic pathway, is attenuated and protective in a murine model of melioidosis. Infect Immun 70:5290–5294. doi:10.1128/IAI.70.9.5290-5294.2002. 12183585 PMC128252

[62] Norris MH, Kang Y, Lu D, Wilcox BA, Hoang TT 2009 Glyphosate resistance as a novel select-agent-compliant, non-antibiotic-selectable marker in chromosomal mutagenesis of the essential genes *asd* and *dapB* of *Burkholderia pseudomallei*. Appl Environ Microbiol 75:6062–6075. doi:10.1128/AEM.00820-09. 19648360 PMC2753064

[63] Norris MH, Kang Y, Wilcox B, Hoang TT 2010 Stable, site-specific fluorescent tagging constructs optimized for *Burkholderia* species. Appl Environ Microbiol 76:7635–7640. doi:10.1128/AEM.01188-10. 20851961 PMC2976199

[64] Norris MH, Propst KL, Kang Y, Dow SW, Schweizer HP, Hoang TT 2011 The *Burkholderia pseudomallei* Δ*asd* mutant exhibits attenuated intracellular infectivity and imparts protection against acute inhalation melioidosis in mice. Infect Immun 79:4010–4018. doi:10.1128/IAI.05044-11. 21807903 PMC3187240

[65] Norris MH 2014 Identifying virulence factors and regulators contributing to pathogenesis by the select-agent bacterium *Burkholderia pseudomallei*. PhD thesis University of Hawaii at Manoa, Honolulu, Hawaii.

[66] Lam JS, Anderson EM, Hao Y 2014 LPS quantitation procedures. Methods Mol Biol 1149:375–402. doi:10.1007/978-1-4939-0473-0_31. 24818921

[67] Pfaffl MW, Horgan GW, Dempfle L 2002 Relative expression software tool (REST) for group-wise comparison and statistical analysis of relative expression results in real-time PCR. Nucleic Acids Res 30:e36. doi:10.1093/nar/30.9.e36. 11972351 PMC113859

[68] Lindner B 2000 Matrix-assisted laser desorption/ionization time-of-flight mass spectrometry of lipopolysaccharides, p 311–325. *In* Holst O (ed), Bacterial toxins: methods and protocols. Humana Press, Totowa, NJ. doi:10.1385/1-59259-052-7:311. 10820729

[69] Choi K-H, Mima T, Casart Y, Rholl D, Kumar A, Beacham IR, Schweizer HP 2008 Genetic tools for select-agent-compliant manipulation of *Burkholderia pseudomallei*. Appl Environ Microbiol 74:1064–1075. doi:10.1128/AEM.02430-07. 18156318 PMC2258562

[70] Kang Y, Norris MH, Wilcox BA, Tuanyok A, Keim PS, Hoang TT 2011 Knockout and pullout recombineering for naturally transformable *Burkholderia thailandensis* and *Burkholderia pseudomallei*. Nat Protoc 6:1085–1104. doi:10.1038/nprot.2011.346. 21738123 PMC3564556

