# Supplementary material for: Lipid A Remodeling Is a Pathoadaptive Mechanism That Impacts Lipopolysaccharide Recognition and Intracellular Survival of Burkholderia pseudomallei
Source: Infect Immun. 2018 Sep 21;86(10):e00360-18. doi: 10.1128/IAI.00360-18 (PMC6204721; doi:10.1128/IAI.00360-18)
Supplement: Supplemental file 1 [file zii999092553s1.pdf]

SUPPLEMENTARY MATERIAL LEGENDS

Figure S1. MALDI scans of lipid A from *lpxO* (A) and *pagL* (B) complemented mutants*.*

Table S1. Percent of lipid A substituents in samples from different temperatures.

Table S2. Percent of lipid A substituents in chronic strains.

Table S3. Percent of lipid A substituents in lipid A modification mutants.
