# Supplementary figures and images for: Lipid A Remodeling Is a Pathoadaptive Mechanism That Impacts Lipopolysaccharide Recognition and Intracellular Survival of Burkholderia pseudomallei
Source: Infect Immun. 2018 Sep 21;86(10):e00360-18. doi: 10.1128/IAI.00360-18 (PMC6204721; doi:10.1128/IAI.00360-18)

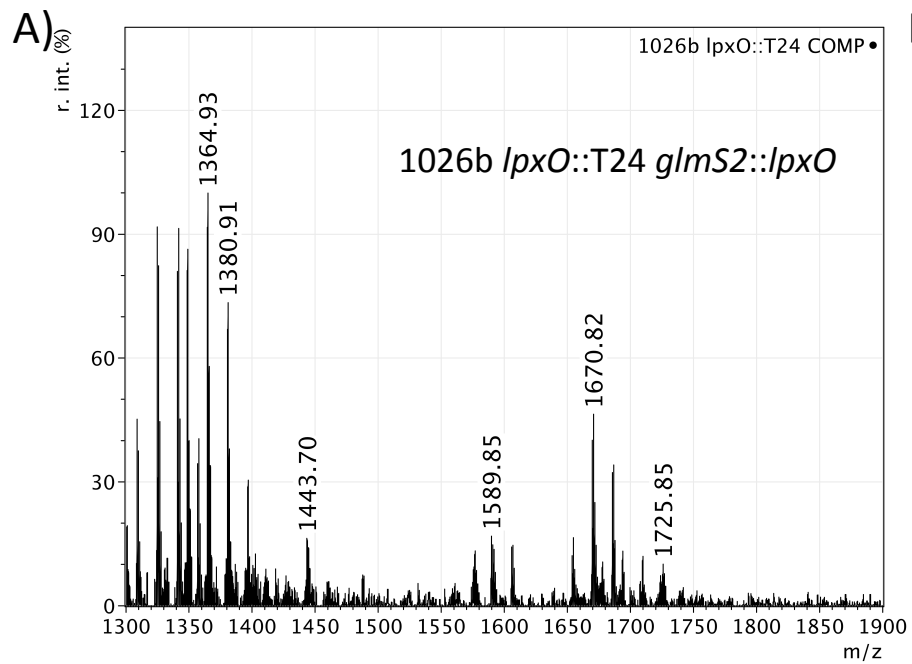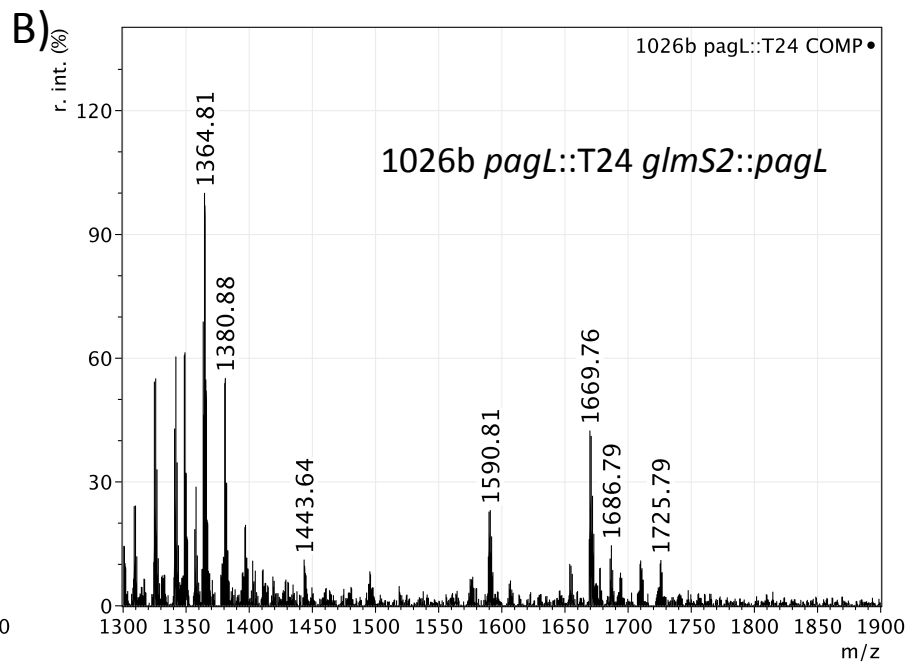

Supplement: Supplemental file 2 [file zii999092553s2.pdf]
