# Supplementary material for: Lipid A Remodeling Is a Pathoadaptive Mechanism That Impacts Lipopolysaccharide Recognition and Intracellular Survival of Burkholderia pseudomallei
Source: Infect Immun. 2018 Sep 21;86(10):e00360-18. doi: 10.1128/IAI.00360-18 (PMC6204721; doi:10.1128/IAI.00360-18)
Supplement: Supplemental file 3 [file zii999092553s3.pdf]

TABLE S1.

Percent of lipid A substituent in samples from different temperatures

Lipid A substituents	Acyl chains	Approx. m/z	% of each lipid A in sample			
			1026b 23°C	1026b 37°C	576a 23°C	576a 37°C
1X 14:0 (3-OH), 2X 16:0 (3-OH), 1X 14:0, 1P	tetra	1365	16.14	45.46	34.04	38.26
1X 14:0 (2-OH), 1X 14:0 (3-OH), 2X 16:0 (3-OH), 1P	tetra	1380	10.29	8.20	13.80	13.32
1X 14:0 (3-OH), 2X 16:0 (3-OH), 1X 14:0, 2P	tetra	1444	2.30	5.25	2.38	2.65
1X 14:0 (2-OH), 1X 14:0 (3-OH), 2X 16:0 (3-OH), 2P	tetra	1460	1.78	2.82	0.85	2.36
	% tetra 2-OH	-	39.55	17.85	28.69	27.71
2X 14:0 (3-OH), 2X 16:0 (3-OH), 1X 14:0, 1P	penta	1590	35.73	8.96	37.83	28.13
1X 14:0 (2-OH), 2X 14:0 (3-OH), 2X 16:0 (3-OH), 1P	penta	1606	13.41	3.93	4.04	5.07
2X 14:0 (3-OH), 2X 16:0 (3-OH), 1X 14:0, 2P	penta	1670	15.05	19.12	6.09	8.97
1X 14:0 (2-OH), 2X 14:0 (3-OH), 2X 16:0 (3-OH), 2P	penta	1686	5.30	6.26	0.97	1.24
	% penta 2-OH	-	26.93	26.62	10.24	14.54
TOTAL	tetra	-	30.51	61.72	51.06	56.58
TOTAL	penta	-	69.49	38.28	48.94	43.42
TOTAL	2-OH	-	30.78	21.21	19.66	21.99
