# Supplementary material for: Lipid A Remodeling Is a Pathoadaptive Mechanism That Impacts Lipopolysaccharide Recognition and Intracellular Survival of Burkholderia pseudomallei
Source: Infect Immun. 2018 Sep 21;86(10):e00360-18. doi: 10.1128/IAI.00360-18 (PMC6204721; doi:10.1128/IAI.00360-18)
Supplement: Supplemental file 4 [file zii999092553s4.pdf]

TABLE S2.

Percent of lipid A substituents in chronic strains

Lipid A substituents	Acyl chains	Approx. m/z	% of each lipid A in sample		
			MSHR1043	MSHR1655	MSHR3042
1X 14:0 (3-OH), 2X 16:0 (3-OH), 1X 14:0, 1P	tetra	1365	30.59	30.82	31.60
1X 14:0 (2-OH), 1X 14:0 (3-OH), 2X 16:0 (3-OH), 1P	tetra	1380	4.16	5.08	2.77
1X 14:0 (3-OH), 2X 16:0 (3-OH), 1X 14:0, 2P	tetra	1444	35.71	26.44	38.36
1X 14:0 (2-OH), 1X 14:0 (3-OH), 2X 16:0 (3-OH), 2P	tetra	1460	4.22	3.16	2.80
1X 14:0 (3-OH), 2X 16:0 (3-OH), 1X 14:0, 1X Ara4N, 1P	tetra	1494	4.35	11.61	9.53
1X 14:0 (2-OH), 1X 14:0 (3-OH), 2X 16:0 (3-OH), 1X Ara4N, 1P	tetra	1510	0.51	2.19	1.22
1X 14:0 (3-OH), 2X 16:0 (3-OH), 1X 14:0, 1X Ara4N, 2P	tetra	1575	3.66	3.21	5.11
TOTAL	tetra	-	83.19	82.50	91.40
2X 14:0 (3-OH), 2X 16:0 (3-OH), 1X 14:0, 1P	penta	1590	4.51	4.78	2.41
1X 14:0 (2-OH), 2X 14:0 (3-OH), 2X 16:0 (3-OH), 1P	penta	1606	0.87	0.68	1.06
2X 14:0 (3-OH), 2X 16:0 (3-OH), 1X 14:0, 2P	penta	1670	10.23	10.97	4.80
1X 14:0 (2-OH), 2X 14:0 (3-OH), 2X 16:0 (3-OH), 2P	penta	1686	1.19	1.07	0.33
TOTAL	penta	-	16.81	17.50	8.60
