# Supplementary material for: Lipid A Remodeling Is a Pathoadaptive Mechanism That Impacts Lipopolysaccharide Recognition and Intracellular Survival of Burkholderia pseudomallei
Source: Infect Immun. 2018 Sep 21;86(10):e00360-18. doi: 10.1128/IAI.00360-18 (PMC6204721; doi:10.1128/IAI.00360-18)
Supplement: Supplemental file 5 [file zii999092553s5.pdf]

TABLE S3.

Percent of lipid A substituents in lipid A modification mutants

Lipid A substituents	Acyl chains	Approx. m/z	% of each lipid A in sample					
			1026b WT	*lpxO*::T24	*pagL*::T24	*B.t. WT*	*lpxO* COMP	*pagL* COMP
1X 14:0 (3-OH), 2X 16:0 (3-OH), 1X 14:0, 1P	tetra	1365	35.81	37.24	4.61	34.21	32.07	38.00
1X 14:0 (2-OH), 1X 14:0 (3-OH), 2X 16:0 (3-OH), 1P	tetra	1380	12.64	-	3.15	-	23.57	20.93
1X 14:0 (3-OH), 2X 16:0 (3-OH), 1X 14:0, 2P	tetra	1444	2.59	7.81	19.97	8.48	5.23	4.21
1X 14:0 (2-OH), 1X 14:0 (3-OH), 2X 16:0 (3-OH), 2P	tetra	1460	1.47	-	6.11	-	-	-
1X 14:0 (3-OH), 2X 16:0 (3-OH), 1X 14:0, 1X Ara4N, 1P	tetra	1494	1.34	5.12	-	8.10	-	-
2X 14:0 (3-OH), 2X 16:0 (3-OH), 1X 14:0, 1P	penta	1590	22.82	23.94	4.29	9.58	5.41	8.77
1X 14:0 (2-OH), 2X 14:0 (3-OH), 2X 16:0 (3-OH), 1P	penta	1606	9.40	-	3.13	-	4.71	2.29
2X 14:0 (3-OH), 2X 16:0 (3-OH), 1X 14:0, 2P	penta	1670	9.80	22.92	38.07	26.87	14.87	16.12
1X 14:0 (2-OH), 2X 14:0 (3-OH), 2X 16:0 (3-OH), 2P	penta	1686	4.13	-	9.98	-	10.93	5.52
2X 14:0 (3-OH), 2X 16:0 (3-OH), 1X 14:0, 1X Ara4N, 1P	penta	1721	-	2.98	1.56	3.67	3.22	4.17
2X 14:0 (3-OH), 2X 16:0 (3-OH), 1X 14:0, 1XAra4N, 2P	penta	1803	-	-	8.19	3.51	-	-
1X 14:0 (2-OH), 2X 14:0 (3-OH), 2X 16:0 (3-OH), 1XAra4N, 2P	penta	1820	-	-	0.94	2.06	-	-
TOTAL	tetra	-	53.84	50.16	33.84	50.79	60.87	63.14
TOTAL	penta	-	46.16	49.84	66.16	45.69	39.13	36.86
TOTAL	2-OH	-	27.65	0.00	22.37	0.00	39.20	28.74
